# Quadrupolar Isotope-Correlation Spectroscopy in Solid-State NMR

**DOI:** 10.1021/acs.jpcc.2c00578

**Published:** 2022-05-17

**Authors:** Tamar Wolf, Michael J. Jaroszewicz, Lucio Frydman

**Affiliations:** Department of Chemical and Biological Physics, Weizmann Institute of Science, Rehovot 7610001, Israel

## Abstract

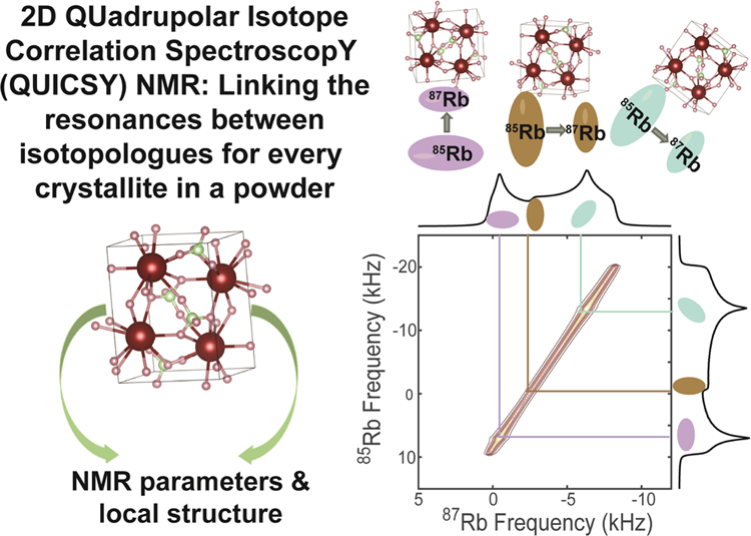

Quadrupolar solid-state NMR carries a wealth of structural information, including insights about chemical environments arising through the determination of local coupling parameters. Current methods can successfully resolve these parameters for individual sites using sample-spinning methods techniques applicable to quadrupolar *I* ≥ 1 nuclei, provided second-order central transition broadenings do not exceed by much the spinning rate. For large quadrupolar coupling (*C*_Q_) values, however, static acquisitions are often preferable, leading to challenges in extracting local structural information. This study explores the use of two-dimensional QUadrupolar Isotope Correlation SpectroscopY (QUICSY) experiments as a means to increase the NMR spectral resolution and enrich the characterization of quadrupolar NMR patterns under static conditions. QUICSY seeks to correlate the solid-state NMR powder line shapes for two quadrupolar isotopes belonging to the same element via a 2D experiment. In general, two isotopes of the same element will have different nuclear quadrupole moments, gyromagnetic ratios, and spin numbers but essentially identical chemical environments. The possibility then arises of obtaining sharp “ridges” in these 2D correlations, even in static samples showing large quadrupolar effects, which lead to second-order line shapes that are several kilohertz wide. Moreover, pairs of quadrupolar isotopes are recurrent in the periodic table and include important elements such as ^35,37^Cl, ^69,71^Ga, ^79,81^Br, and ^85,87^Rb. The potential of this approach is explored theoretically and experimentally on two rubidium-containing salts: RbClO_4_ and Rb_2_SO_4_. We find that each compound gives rise to distinctive 2D QUICSY line shapes, depending on the quadrupolar and chemical shift anisotropy (CSA) parameters of its sites. These experimental line shapes show good agreement with analytically derived 2D spectra relying on literature values of the quadrupolar and CSA tensors of these compounds. The approach underlined here paves the way toward better characterization of wideline NMR spectra of quadrupolar nuclei possessing different nuclear isotopes.

## 1 Introduction

The NMR of solids provides a valuable understanding of the local structure and dynamics of a broad range of inorganic and biological materials. Half-integer quadrupolar nuclei provide essential probes in these characterizations as they constitute the majority of the NMR-active elements^1^ and make up key materials including biominerals, glasses, ceramics, and solid electrolytes; quadrupolar *I* ≥ 1 species are often also at the reactive centers of biochemical events.^2,3^ The NMR of these species is often dominated by the quadrupolar interaction, arising from the electrostatic coupling between the quadrupolar moment of these nuclei and an electric field gradient (EFG). The EFG is defined by the electronic environment surrounding the species in the solid; it carries rich structural information and is highly sensitive to changes in local atomic environments.^1,2,4^ Besides these advantages, quadrupolar effects can bring about substantial anisotropic broadenings, challenging the elucidation of NMR spectra on polycrystalline samples and precluding a clear interpretation of overlapping patterns in the presence of multiple chemical sites. For this reason, quadrupolar NMR is often circumscribed to half-integer *I* ≥ 3/2 spins, which presents a relatively narrow central transition (CT). These −1/2 ↔ +1/2 CTs will be solely affected by quadrupolar broadenings up to the second order, yielding linewidths of the order of *C*_Q_^2^/*ω*_0_, where *C*_Q_ is the quadrupolar coupling constant and *ω*_0_ is the spin’s Larmor frequency. As second-order effects can challenge the acquisition of solid-state quadrupolar NMR spectra numerous routes to improve the interpretation and resolution of CT spectra have been developed. These include methods that rely on multiple sample-spinning axes^5−9^ as well as on fixed spinning at the magic angle of 54.7°, but call for 2D correlations between central and multiple-quantum (MQMAS) or between central and satellite (STMAS) transitions.^10−14^ Although these experiments are widely used to study materials,^2,11−15^ they may still fail when tackling quadrupolar sites characterized by large *C*_*Q*_ values, particularly when the CT second-order broadenings are so large that they substantially exceed the rates of MAS sample spinning. Species characterized by a large *C*_Q_ are therefore best measured under static, low-resolution conditions,^16−19^ thus preventing the sensitivity losses associated with MQMAS/STMAS as well as the numerous overlapping spinning sidebands that may otherwise arise. Approaches to discern quadrupolar-broadened sites under static conditions have thus been discussed based on variable-field acquisitions^20^ and on differing nutation behaviors;^21^ the use of multi-quantum 2D correlations to enrich the information of static NMR has also been explored.^22^ This study discusses an alternative route based on 2D correlations.

A common characteristic of spinning-based 2D NMR experiments used for resolving quadrupolar second-order powder patterns is a reliance on two consecutive periods where spins are subject to anisotropic evolutions that are, in some way, “proportional” to one another for every crystallite in the sample. It is owing to this proportionality that echoes refocusing the residual quadrupolar broadening arise in the (*t*_1_, *t*_2_) time domain. In dynamic-angle spinning,^8,9^ a judicious choice of the two different spinning axes and evolution times imparts a proportionality that aligns the various CT powder line shapes along (*F*_1_,*F*_2_); in MQMAS and STMAS experiments, the proportionality arises from Clebsch–Gordan coefficients, which make the evolution of the anisotropies left over by the MAS proportional to one another for every crystallite in the powder. Similarly, this study was motivated by the realization that a certain “proportionality” between the anisotropic quadrupolar broadenings could be imposed if correlations were performed among CTs of different isotopes of the same element: quadrupolar second-order shifts in such isotopes will be given by EFG properties that are virtually independent of the nature of the isotope, modulated by orientation-independent scalar parameters, i.e., gyromagnetic ratios, spin numbers, and nuclear quadrupole moments, which scale the strength of the interaction. In such cases, the possibility could arise of yielding one-to-one correlations for the CT frequencies of every crystallite in a powder so as to arrive at high-resolution 2D correlations even in the absence of MAS. On the other hand, in the presence of multiple magnetically or crystallographically (chemically) inequivalent sites, such powdered 2D correlations should feature off-diagonal peaks containing rich information. Moreover, quite a few elements in the periodic table possess two quadrupolar isotopes in relatively high abundances, including ^35,37^Cl, ^69,71^Ga, ^79,81^Br, ^63,65^Cu, ^135,137^Ba, and ^85,87^Rb; out of these, halogens are often present in active pharmaceutical ingredients,^23,24^ whereas species such as Ga are present in semiconductors and metal–organic frameworks.^25,26^

Based on such considerations, the present study explores static NMR versions of what we denominate as QUadrupolar Isotope Correlation SpectroscopY (QUICSY), a 2D experiment correlating the CTs of different quadrupolar isotopes of the same element. To this end, we treat a number of theoretical scenarios pertaining to static spectra acquired on nuclei broadened by second-order quadrupolar effects: first in the absence and then in the presence of the chemical shift interaction. Following these considerations, we explore the static 2D QUICSY approach on the isotope pair ^85^Rb and ^87^Rb for compounds bearing one and two chemically inequivalent crystallographic sites. For each case, a unique pattern is achieved containing rich information regarding the interaction tensors and their relative orientations. The experimental results arising from these considerations matched well with analytical 2D calculations based on existing literature parameters, validating the use of theoretical arguments to estimate the benefits and insight arising from this approach.

## 2 Materials and Methods

### 2.1 Samples

Polycrystalline RbClO_4_ and Rb_2_SO_4_ (Strem Chemicals, 99.9%) were used as received, after grinding them into fine powders and packing into 4 mm zirconia NMR rotors, for measurement under static conditions. In these samples, the natural abundance of ^85^Rb is 72.17% and that of ^87^Rb is 27.83%.

### 2.2 Solid-State NMR Spectroscopy

NMR experiments were performed using a Varian VNMRS console interfaced to an Oxford 14.1 T (ω_0_(^1^H) = 600 MHz) wide-bore magnet. A Varian 4 mm triple-resonance HXY probe was used for this study, with the X and Y channels tuned to the high- and low-frequency-correlated isotopes, respectively. Chemical shift referencing was performed for ^87^Rb and ^85^Rb using a dilute RbNO_3_ solution. Pulse-width calibrations were performed on solid RbBr: directly for ^87^Rb and indirectly for ^85^Rb, which was calibrated via its cross-polarization (CP) to ^87^Rb. All used sequences relied on such ^85^Rb → ^87^Rb CPs and included either a 1D CP^27,28^ with Carr–Purcell–Meiboom–Gill (CPMG) acquisition^29−33^ or 2D constant-time^34−36^ CP-CPMG with whole-echo acquisition for an absorptive line shape.^37,38^ The phase-cycling used for the 1D CP-CPMG was as follows: ϕ_1_ = *x*,–*x*, ϕ_3_ = *y*, ϕ_4_ = *x*,*x*,–*x*,–*x*,*y*,*y*,–*y*,–*y*, ϕ_4_ = *x*,–*x*,*x*,–*x*,*y*,–*y*,*y*,–*y*, and ϕ_rec_ = ϕ_1_ + ϕ_4_ = *x*,–*x*,–*x*,*x*,*y*,–*y*,–*y*,*y*. The phase-cycling of the 2D constant-time CP-CPMG was altered to select a single CT-detected evolution pathway in the indirect dimension: ϕ_1_ = *x*,*y*,–*x*,–*y*, ϕ_2_ = 4*{*x*}, 4*{*y*}, 4*{−*x*}, 4*{−*y*}, ϕ_3_ = 16*{*x*}, 16*{*y*}, 16*{−*x*}, 16*{−*y*}, ϕ_4_ = *x*, ϕ_5_ = *x*, and ϕ_*rec*_ = ϕ_1_ – 2ϕ_2_ + ϕ_3_. In all cases, a converging double-frequency sweep (DFS) pulse was applied on the ^85^Rb channel prior to the ^85^Rb → ^87^Rb CP for signal enhancement,^55,56^ with the following parameters: 1.9 ms duration; initial/final frequencies (from the CT center): 6600/200 kHz; number of steps: 27,000; and RF amplitude: 20 kHz. The same DFS parameters were used for both Rb_2_SO_4_ and RbClO_4_. For processing the 2D CP-CPMG QUICSY data, CPMG echoes were spliced and co-added in the direct dimension. Unless otherwise specified, a symmetric whole echo was also collected in the *t*_1_-domain. As no dispersive component arises from such fully *t*_1_/*t*_2_ echoed acquisitions, all 2D data are presented in the magnitude mode. No line-broadening was added, and a zero-filling of 2048 points in *F*_2_ and 512 points in *F*_1_ was used. For more details, see the following text and Supporting Information.

### 2.3 Simulations

All calculations/simulations focused on the CT of half-integer quadrupolar nuclei. Analytical calculations to describe 2D correlation spectra of two isotopes affected by second-order quadrupolar interactions and by isotropic/anisotropic chemical shifts (CSA) were coded in MATLAB. All of these 2D spectral calculations assumed ideal correlations among the evolution frequencies of the two isotopes for each crystallite orientation: i.e., equal efficiencies for the information transferred during the mixing. The orientation-dependent CSA effects were calculated on powdered samples by first transforming their tensors from a CSA principal axis system (PAS) to the PAS of the EFG tensor and then onto the laboratory frame. In the case of two or more magnetically inequivalent sites per crystallographic unit, an additional transformation was added to relate the PAS of one of the EFG tensors to the PAS of the other EFG tensor. All transformations and conventions used are described in the Appendix. The full expressions of the orientation dependencies were calculated with Mathematica,^39^ and the accuracy of these lab-written codes was verified by comparisons against 1D numerical simulations arising from the Simpson^40^ programming software (not shown). Throughout the text and the Supporting Information, frequency-based calculations were used; for comparisons with the experimental data, equivalent time-domain calculations were performed to obtain comparable spectral resolutions upon processing. All MATLAB scripts are available upon request; the Mathematica scripts used in this study can also be found in the Supporting Information.

## 3 Theoretical Background

**Figure 1:**
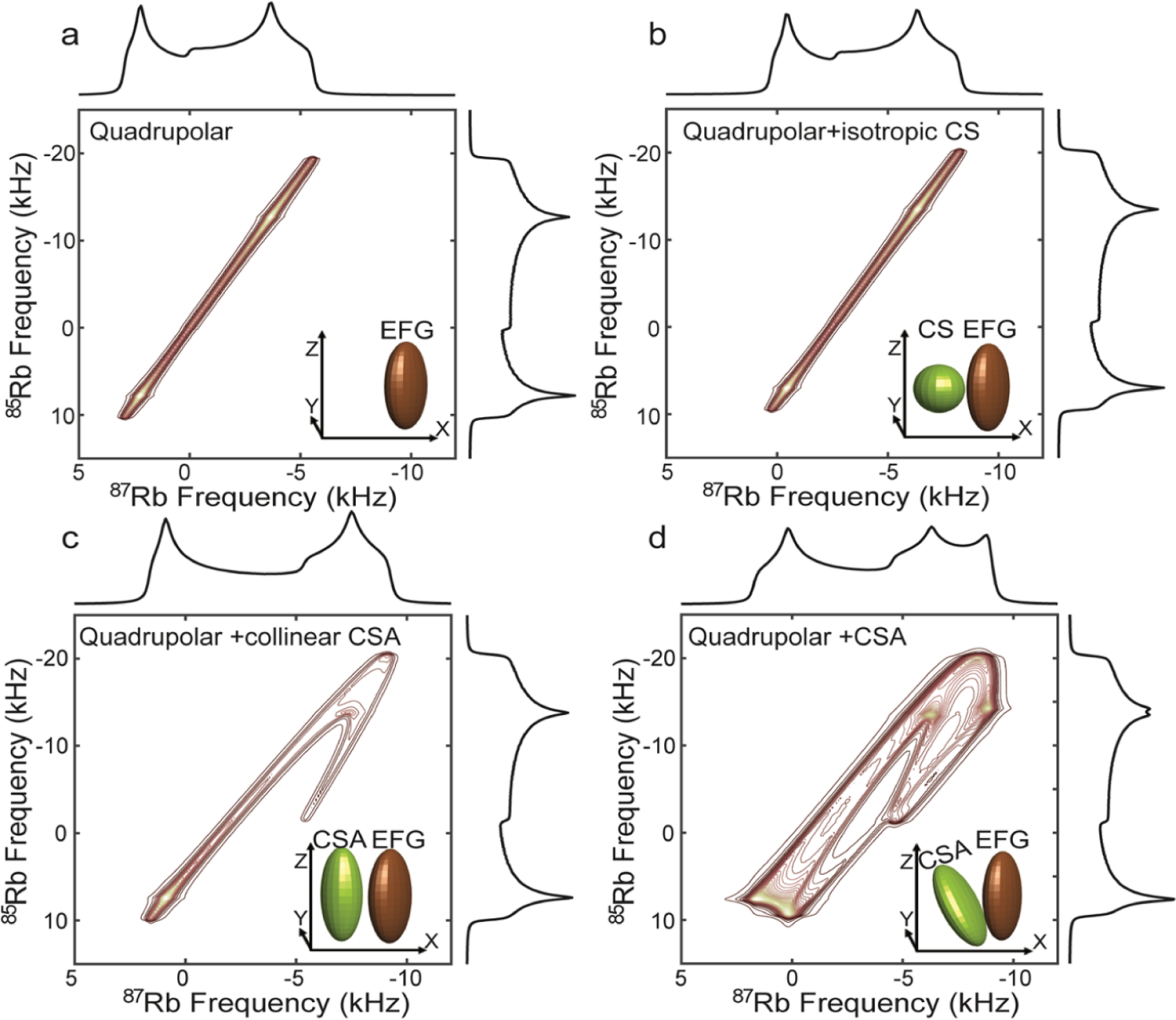
Calculated 2D QUICSY spectra of a single site affected by either a second-order quadrupolar interaction alone (a) or by both chemical shift and quadrupolar interactions (b–d). (a) 2D QUICSY for an ^85^Rb/^87^Rb isotope pair subjected solely to a quadrupolar interaction with *C*_Q_ = 3.3 MHz and η_Q_ = 0.21 for ^87^Rb (ω_0_ = 196.26 MHz). The *C*_Q_ of ^85^Rb was scaled by the ratio of quadrupole moments of the nuclei.^41^ (b) The same as in panel (a) but upon introducing an isotropic chemical shift δ_iso_ = −13.7ppm. (c) The same as in panel (b) but upon introducing a CSA with δ_aniso_ = −13.8 ppm. The CSA tensor is collinear with the quadrupolar tensor and is axially symmetric, i.e., η_CS_ = 0. (d) The same as in panel (c) but for a non-coincident CSA tensor with η_CS_ = 0.61, ψ = 94°, χ = 28°, and ξ = 87° (i.e., the literature-given quadrupole and chemical shift anisotropy parameters of RbClO_4_^45,46^). The insets depict pictorially the relative orientation of the PAS of the quadrupolar tensor (brown ellipsoid) and the CSA tensor (green ellipsoid).

This study considers two different isotopes of the same element; each of these is a half-integer quadrupolar species described by a distinct spin *I*, gyromagnetic ratio γ defining both the Larmor frequency ω_o_ and the chemical shift ω_cs_, and nuclear quadrupole moment *eQ*. Disregarding isotope effects, one can assume that for a certain compound, these two isotopes will be subject to identical surroundings, and thus their EFG and shielding anisotropy will, for a given magnetically and chemically equivalent site, be the same. In consequence, for a given crystallite orientation, the Hamiltonians of the two isotopes will be affected by the same orientation-dependent terms.^41^ Their difference will thus be given by *eQ* and γ-driven scalings of their quadrupolar and chemical shift frequencies, respectively. Figure 1 presents a number of scenarios that will then arise when considering 2D QUICSY correlations, taking the ^85^Rb and ^87^Rb isotopes as prototypical examples. In these cases, the correlated frequencies will be given by the orientation-dependent frequency of each isotope’s central transition
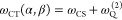
1where the second-order quadrupolar shift is given by^42^ (see Appendix)
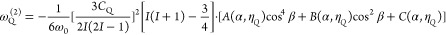
2and the chemical shift is represented as^41,43,44^
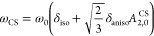
3In these equations, α and β are Euler angles that define the orientation of the static magnetic field in the PAS of the EFG tensor. The CSA and quadrupolar interaction tensors need not be coincident and, in general, will have their respective PASs related by Euler angles ((ψ,χ,ξ); see Appendix for more details). For sites that are affected solely by a quadrupolar interaction (ω_CS_ = 0), the result of such an inter-isotope correlation would be a narrow ridge, even for a powder pattern (see Figure 1a). The proportionality constant between the second-order quadrupolar effects of the correlated CTs will dictate the slope of the ensuing ridge; on the basis of eq 2, it will be
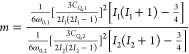
4Here, the subscripts 1 and 2 denote the two isotopes being correlated, assumed in Figure 1 to involve ^87^Rb and ^85^Rb, respectively. Adding an isotropic chemical shift does not affect the high resolution of these correlations but only shifts the ridge in both dimensions (Figure 1b). Under these premises, multiple sites with different isotropic chemical shifts could in principle still be resolved. The addition of a collinear, axially symmetric chemical shift anisotropy interaction alters the proportionality between the *F*_1_ and *F*_2_ frequencies over the powder; yet, as long as η_Q_ is close to zero, the correlation retains a narrow parabola-like contour (Figures 1c and S1). Upon considering axially asymmetric and noncoincident quadrupolar/CSA tensors, however, the nature of the 2D correlation is further altered and the narrow ridge becomes broader, with the exact shape of this contour containing a wealth of information regarding the coupling parameters. Figure 1d depicts such a line shape for a case based on RbClO_4_;^45^ the contour’s dependence on the relative orientation and symmetry of the two interaction tensors is further illustrated in Supporting Figure S1.

**Figure 2:**
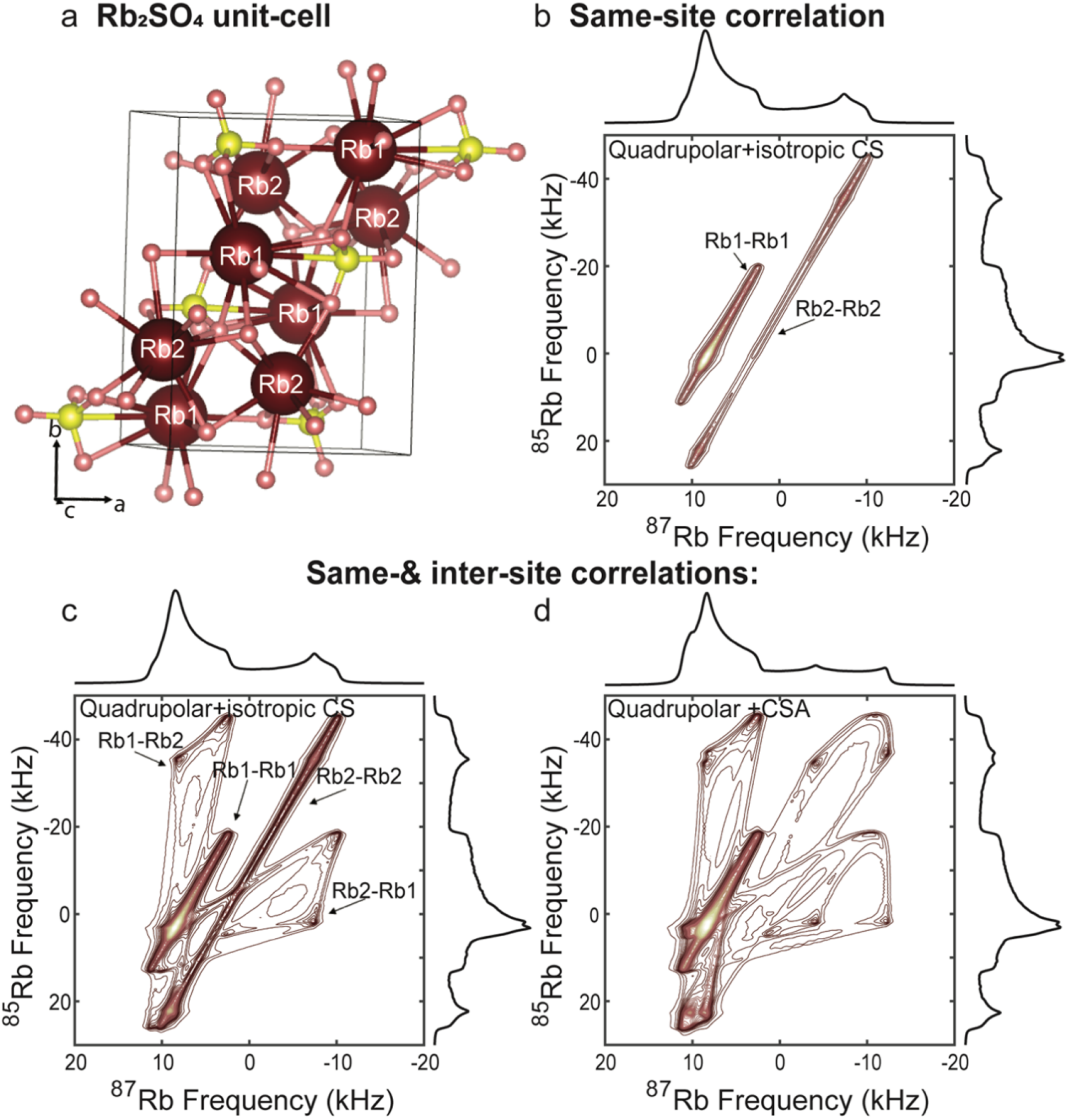
Calculated 2D QUICSY spectra of two crystallographic sites inspired by Rb_2_SO_4_. (a) Orthorhombic structure of Rb_2_SO_4_ (space-group *Pnam*(47)), with the Rb atoms displayed in brown and marked as Rb1 or Rb2 to describe the two Rb sites, oxygen atoms in light pink, and S atoms in yellow (image generated with the VESTA program^48^). (b) 2D QUICSY for two ^85^Rb/^87^Rb pairs, characterized by intrasite correlations: Rb1–Rb1 and Rb2–Rb2. The two sites are affected by the quadrupolar interaction and isotropic chemical shift of the following parameters for ^87^Rb: *C*_Q,1_=2.72 MHz, η_Q,1_ = 0.93, δ_*iso*,1_ = 42.6 ppm; *C*_Q,2_=5.29 MHz, η_*Q*,2_ = 0.12, δ_iso,2_ = 15.5 ppm. (c) 2D QUICSY including same and inter-site correlation terms between the two sites, with equal probabilities: Rb1–Rb1, Rb1–Rb2, Rb2–Rb1, and Rb2–Rb2. (d) The same as in panel (c) but with the addition of the following CSA parameters: δ_aniso,1_ = 2.7 ppm, η_CS,1_ = 0.26, ψ_1_ = 76°, χ_1_ = 17°, ξ_1_ = 110°; δ_aniso,2_ = −25 ppm, η_CS,2_ = 0.54, ψ_2_ = 9°, χ_2_ = 37°, and ξ_2_ = 270°. The parameters taken are based on the literature values of Rb_2_SO_4_.^45,49^

When approaching systems with multiple chemical sites (Figure 2a), the situation becomes more complex as both same-site and inter-site correlations are possible. Same-site correlations refer to situations where the cross-peaks between the different isotopes arise from a single magnetically (and thereby chemically) equivalent site in the unit cell. Although such same-site correlations will still retain a high resolution (Figure 2b), isotopological correlations among inequivalent sites will not preserve the high resolution. They will, however, lead to distinctive patterns containing potentially valuable information about the quadrupolar and CSA components of each site as well as the tensors of the two sites (Figure 2b,d). The probability of all of these correlations is expected to scale according to the dipolar interaction strength among the sites, scaled by the polarization transfer efficiency between the two isotopes.

**Figure 3:**
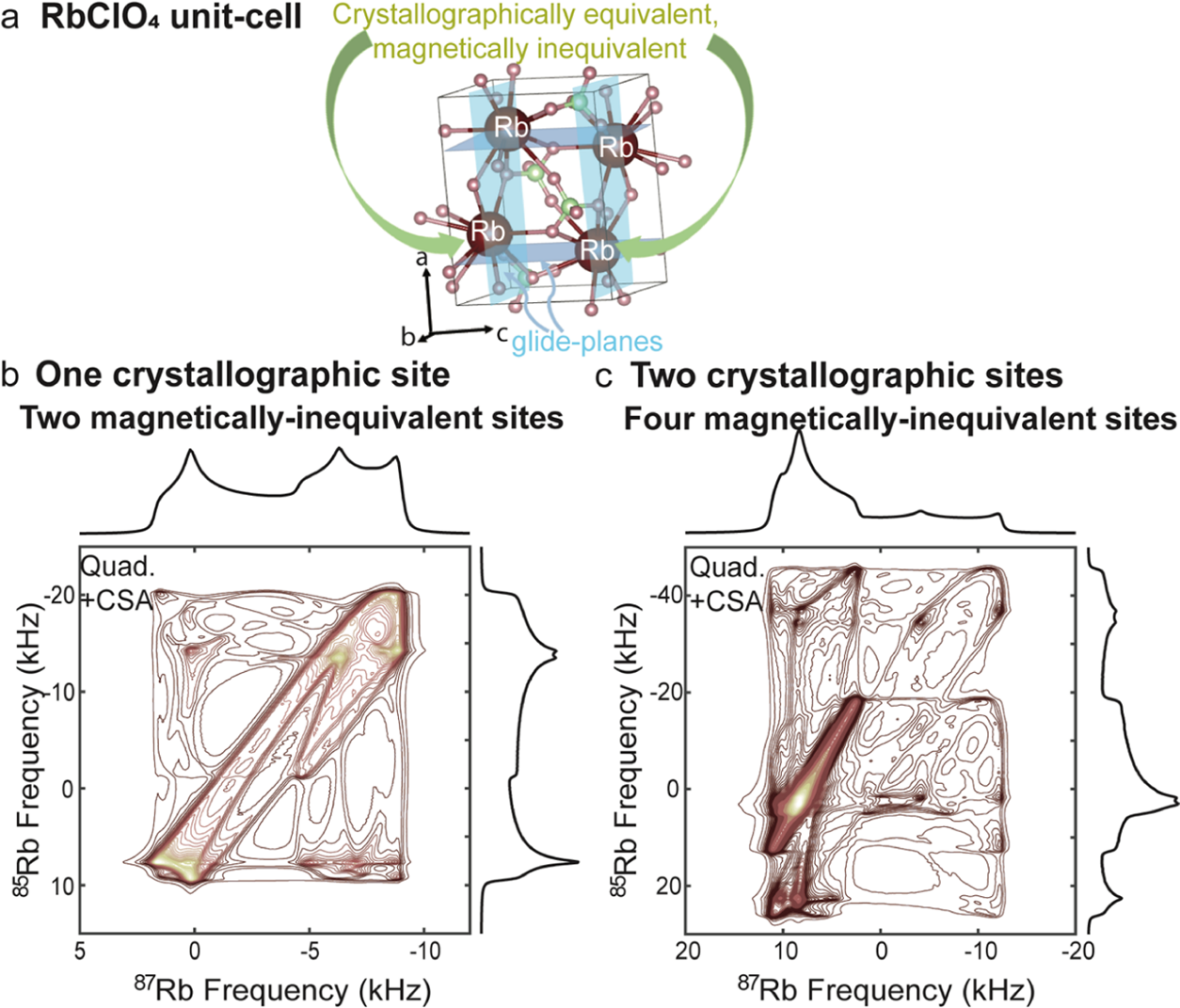
Calculated 2D QUICSY spectra of magnetically inequivalent sites inspired by RbClO_4_. (a) Orthorhombic structure of RbClO_4_ (space-group *Pnma*(53)), with the Rb atoms displayed in brown, oxygen atoms in light pink, and Cl atoms in green (image generated with the VESTA program^48^). Pairs of crystallographically equivalent yet magnetically inequivalent Rb atoms are related by glide planes (shown in light blue); the NMR tensors of these sites are related by a symmetry operation. (b, c) Analytical calculations of 2D correlation experiments between ^85^Rb and ^87^Rb with two magnetically inequivalent sites for each crystallographic site. (b) 2D spectrum of a crystallographic unit consisting of two magnetically inequivalent sites, with a relative orientation of ϕ = −112°, κ = 103°, and ζ = 24° based on the single-crystal data of RbClO_4._^45^ Other NMR parameters are identical to those shown in Figure 1d. (c) 2D spectrum with two crystallographic sites, each of them consisting of two magnetically inequivalent sites with relative orientations of ϕ_1_ = 84°, κ_1_ = 174°, and ζ_1_ = −100° and ϕ_2_ = 39°, κ_2_ = 43°, and ζ_2_ = 97°, based on the single-crystal data of Rb_2_SO_4_ (see Appendix for details of the calculation).^45^ Other NMR parameters are as shown in Figure 2d.

Even when dealing with chemically identical sites, crystallographic symmetry operations such as reflections or glide planes may still lead to magnetically inequivalent sites; this is illustrated for the RbClO_4_ unit cell in Figure 3a.^45,50−52^ Here, all four Rb atoms in the unit are crystallographically equivalent, but they are composed of two pairs of magnetically inequivalent Rb atoms related by glide planes perpendicular to the *a* and *c* crystallographic axes.^45^ It follows that two different orientation-dependent frequencies are present per single crystallite orientation, leading to single-site QUICSY correlations such as the one shown in Figure 3b. It should be noted that the relative orientations of the CSA and quadrupolar tensors remain the same for the two magnetically inequivalent sites since both tensors are related by the same symmetry operations.^45^ The ensuing correlations will contain in this case a redundancy regarding the relative orientation of the quadrupolar tensors of the chemically identical yet magnetically inequivalent sites, of the type that typically arises in single-crystal NMR measurements.^50^ Even further features will arise if chemically as well as magnetically inequivalent sites are present in the unit cell, as illustrated in Figure 3c.

## 4 Results and Discussion

**Figure 4:**
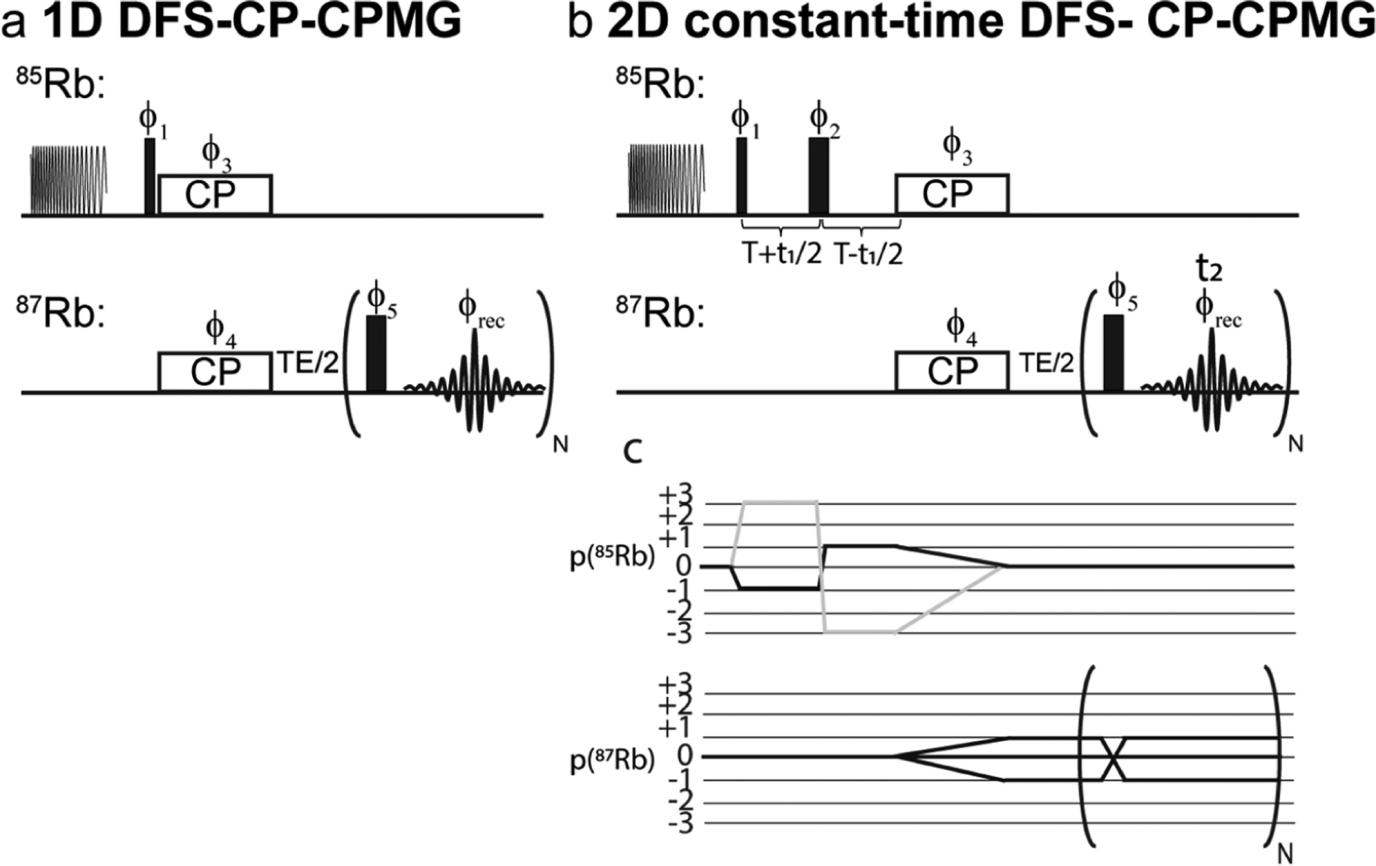
Sequences used in this study. (a) 1D CP-CPMG acquisition preceded by a double-frequency sweep (DFS) block on ^85^Rb for signal enhancement. (b) 2D QUICSY sequence involving a constant-time CP-CPMG with whole-echo acquisitions in *t*_1_ and *t*_2_, preceded by a DFS block (see Materials and Methods for the phase-cycling employed). (c) Coherence transfer pathways for the sequence in (b): after the first excitation pulse on ^85^Rb, a constant-time acquisition enables the sampling of a full SQC echo in *t*_1_, which is then transferred by CP to ^87^Rb. On ^87^Rb, only a single (−1) SQC is then detected throughout a CPMG train. Note that the four-step phase-cycling executed in *t*_1_ will also allow a Δ*p* = + 3 (marked in light gray) to evolve, but this contribution is likely negligible under static conditions.

**Figure 5:**
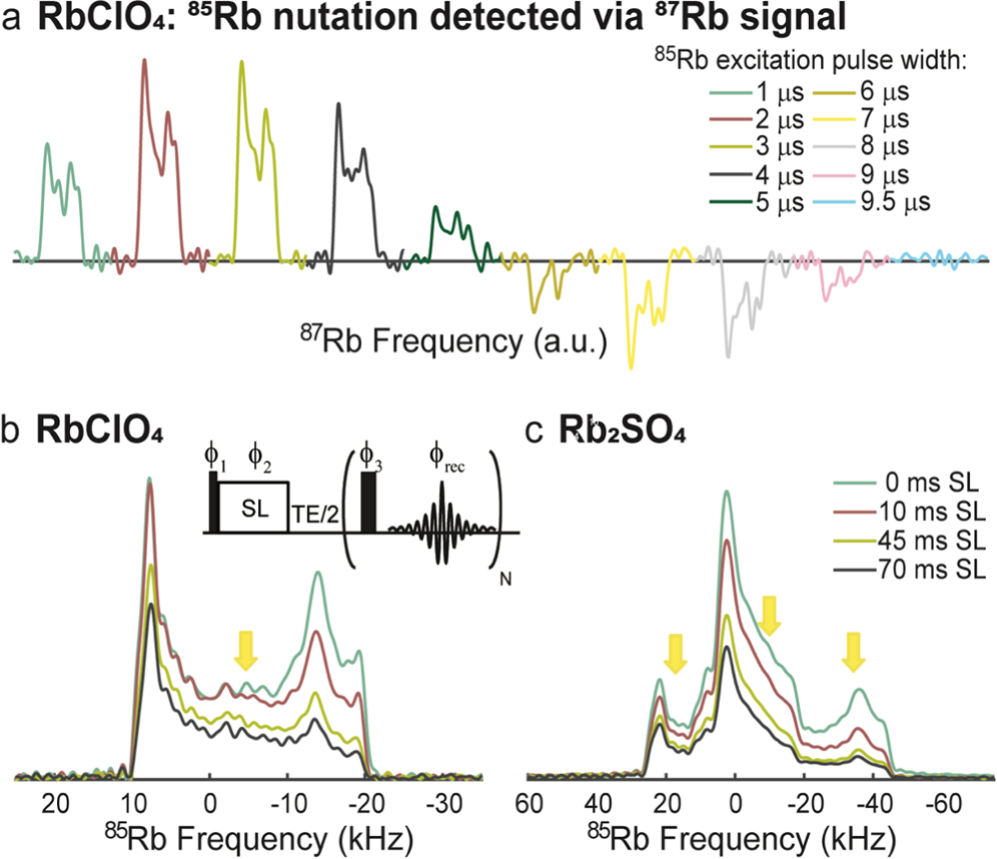
(a) 1D CP-CPMG nutation experiment on RbClO_4_, showing that the phased CPMG signal of ^87^Rb followed the ^85^Rb nutation. The contact time for CP was 45 ms, with an RF amplitude of ca. 30 kHz on the ^85^Rb channel and 45 kHz on the ^87^Rb channel. (b,c) ^85^Rb CT line shapes and intensities as a function of the spin-lock (SL) duration given in milliseconds for (b) RbClO_4_ and (c) Rb_2_SO_4_ using an RF amplitude of ca. 26 kHz. The Rb_2_SO_4_ spectrum is a sum of sub-spectra collected at three different transmitter frequency offsets (18, −10, and −35 kHz), marked by yellow arrows (for the separate sub-spectra, see Figure S2). The RbClO_4_ spectrum was collected at a single frequency offset marked by a yellow arrow. The acquisition was carried out with CPMG to overcome the dead time for ^85^Rb. The phase-cycling employed was ϕ_1_ = *x*,*x*,–*x*,–*x*,*y*,*y*,–*y*,–*y*, ϕ_2_ = −*y*,–*y*,*y*,*y*,*x*,*x*,–*x*,–*x*, ϕ_3_ = *y*,–*y*,*y*,–*y*,*x*,–*x*,*x*,–*x*, and ϕ_*rec*_ = *x*,*x*,–*x*,–*x*,*y*,*y*,–*y*,–*y*.

Figure 4 presents pulse sequences developed to test the 2D QUICSY correlation experiment. To tune the experiment, a 1D CP-CPMG sequence (Figure 4a) was utilized to find the optimal DFS pulse,^54−56^ and good CP matching conditions linking the CTs of the two Rb isotopes. Given the different spin numbers of the Rb isotopes, I(^87^Rb) = 3/2 and I(^85^Rb) = 5/2,^57,58^ and the selective ω_1_ ≪ ω_*Q*_ irradiation conditions under which experiments were performed, optimal CP was found when 2ω_1_(^87^Rb) ≈ 3ω_1_(^85^Rb). After suitable tuning, varying the flip angle of ^85^Rb clearly reflected in the phased CPMG signal of the ^87^Rb spectrum, verifying the direct correlation of both isotopes (Figure 5a). Notice that complexities associated with CP between half-integer quadrupoles undergoing MAS^57,59−61^ will be absent under QUICSY’s static conditions, which are compatible with conventional CP transfer protocols. Still, bandwidth and relaxation limitations may arise, particularly given the short T_1_s and relatively low B_1_s for low-γ nuclei such as ^85^Rb. As a part of this study, we tested the effect of these factors by monitoring the ^85^Rb CT line shapes and intensities following spin-lock for both RbClO_4_ and Rb_2_SO_4_ under static conditions (Figures 5b,5c, and S2). During a spin-locking pulse applied on the ^85^Rb channel at a single offset, the line shape of ^85^RbClO_4_ was slightly altered as a function of the pulse length, but its main features were preserved. The rapid anisotropic relaxation of ^85^Rb (in RbClO_4_ T_1_(^85^Rb) = 80 ms^41^) also meant that after 70 ms of spin-lock, a reduction in the intensity and some distortions became visible. The limited CP bandwidth problem could be overcome by frequency-stepped acquisitions: a Rb_2_SO_4_ pattern arising from the summation of CP traces collected using three different offsets on the ^85^Rb channel yielded a faithful preservation of the overall line shape (Figures 5c and S2).^62−65^ In contrast, owing to the longer T_1_ and T_1ρ_ (T_1_(^87^Rb) = 210–220 ms^41,46^), the spin-lock on ^87^Rb maintained the line shapes for all of the compounds examined in this study (Figure S3). It should be noted that reverting the direction of the transfer used, i.e., going from ^87^Rb to ^85^Rb, resulted in diminished efficiency; this is likely due to the short T_1_ and T_1ρ_ of ^85^Rb,^41^ which were in the order of the contact times used. It is worth noting that other variants for establishing this kind of correlation were also tested, including intermediate transfer through ^1^Hs for protonated compounds. Although some of these proved feasible, the regular CP version described above appeared to be the most advantageous option in terms of the signal-to-noise ratio (SNR) for all of the compounds studied (Figures S4 and S5).

With the optimization of the heteroisotopic correlation thus established, constant-time 2D experiments incorporating the DFS, the CP, and a CPMG acquisition block to improve the SNR (Figure 4b) were executed. Whole-echo acquisitions were utilized for obtaining absorptive 2D line shapes as they yielded higher sensitivities than hypercomplex (or States^38^) acquisitions; although the whole-echo CPMG t_2_ acquisitions meant that full t_1_ echoes were not needed for avoiding 2D mixed-phase line shapes, we found whole-echo t_1_ acquisitions advantageous sensitivity-wise. (see Supporting Figure S6). All 2D spectra are thus presented in magnitude mode. The phase-cycling for these 2D correlation experiments included a full four-step nested phase cycle of the first three pulses ϕ_1_–ϕ_3_ (for a total 64-step phase-cycling) to select a single SQC pathway on ^85^Rb (see Materials and Methods and Figure 4b,c).

**Figure 6:**
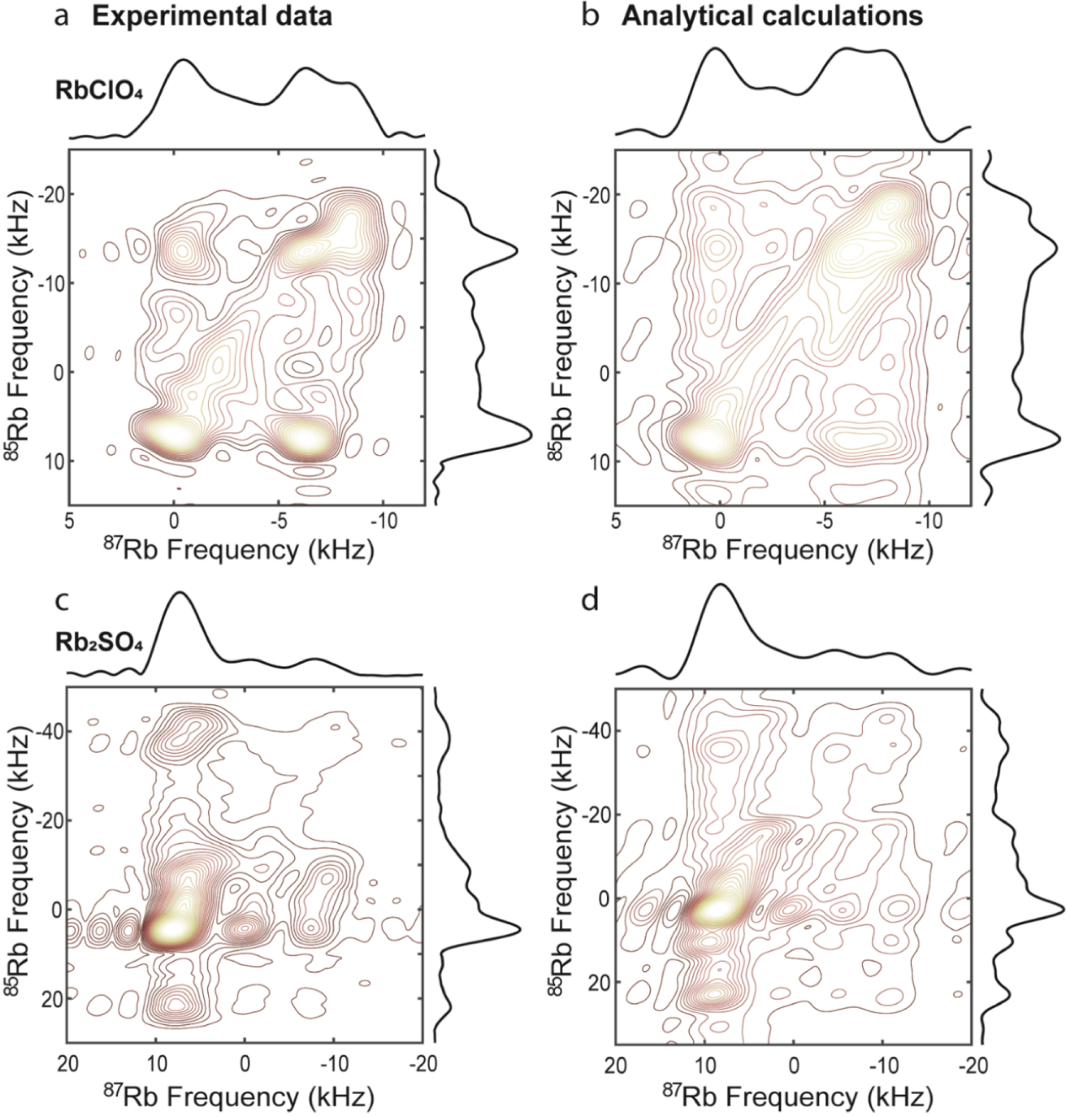
Experimental and calculated 2D QUICSY spectra of RbClO_4_ (a, b) and Rb_2_SO_4_ (c, d). (a) Experimental parameters: CP contact time of 45 ms; CP RF amplitude ∼30 kHz on the ^85^Rb channel and ∼45 kHz on the ^87^Rb channel; whole-echo acquisition performed with the CPMG echo time TE = 720 μs (7 μs dead time before and after each π pulse, sw = 100 kHz); and a total of 23 t_1_ increments sampling a symmetric t1 echo (sw1 = 50 kHz). The SNR of the lowest contour level is 7. (b) Analytical calculation with the same parameters as in Figure 3b. The calculation was performed with 36 points in *F*_2_ (sw = 100 kHz) and 12 points in *F*_1_ (sw_1_ = 50 kHz). (c) Experimental QUICSY spectrum of Rb_2_SO_4_ acquired at three different ^85^Rb offsets (−35, −10, and 18 kHz) and subsequently summed up (see Figure S7). The 2D spectrum was acquired with a CP contact time of 70 ms and the same matching conditions as those for RbClO_4_. The CPMG echo acquisition time was TE = 320 μs (7 μs dead time before and after each π pulse, sw=100 kHz) and a total of 49 *t*_1_ increments constituting a symmetric t1-echo (sw1 = 150 kHz). The SNR of the lowest contour lever is 12. It is possible to collect a slightly asymmetric t1-echo without compromising the 2D contour as well as to reduce sw1 (Figure S8). (d) Analytical calculation with literature parameters of Rb_2_SO_4_ identical to those in Figure 3c. Simulations used 16 points in *F*_2_ (sw = 100 kHz) and 25 points in *F*_1_ (sw_1_ = 150 kHz).

Figure 6a,c shows representative 2D QUICSY spectra of RbClO_4_ and Rb_2_SO_4_. The experimental 2D spectra bear a close resemblance to analytically calculated 2D correlations based on the literature values despite the fact that the calculated spectra disregard the inefficiencies and heterogeneities of the DFS, CP, or CPMG processes (Figure 6b,d). As for the literature values employed, different sources list somewhat different chemical shift parameters, particularly with regard to the relative chemical shift tensor orientation (Tables S1 and S2). A good match was found between the experimental 2D QUICSY spectrum of RbClO_4_ and the literature set in ref (45), with the exception that δ_*aniso*_’s sign had to be reversed for achieving this (δ_aniso_ = −13.8 ppm; a negative value of δ_aniso_ was also reported in a previous study^46^). It can be seen that the experimental spectrum is slightly asymmetric as compared with the ideal analytical calculation; this could reflect the offset-dependent CP efficacy mentioned earlier. Differences between experimental and calculated spectra may also arise due to the simplified assumption of equal probability for all transfers regardless of orientation; still, differences between experiments and analytical expectations are too small to enable their refinement. Notice how the extensive cross-peak structure in the RbClO_4_ spectrum clearly indicates more than one magnetically inequivalent site per single crystallographic unit (Figure 3); this is a type of information that arises in homonuclear correlations^66,67^ and in single-crystal NMR, but it is not usually available from correlations among different NMR species. Rb_2_SO_4_ 2D QUICSY experiments acquired at three different ^85^Rb offsets (Figure 6c, see Supporting Figure S7 for the separate 2Ds) also show a clear fine structure. Literature sources differ somewhat with regard to the chemical shift parameters and orientation of the two different sites of Rb_2_SO_4_ (Table S2); however, again, our data show a good match with the literature values in ref (45) (Figure 6d). Figures S9 and S10 further explore this potential by providing difference maps between the theoretical and experimental data as well as a fitting procedure attempting to extract the coupling parameters from the RbClO_4_ data, respectively. From these and other tests, we conclude that although QUICSY can be a useful tool for extracting this kind of tensorial information, dealing with multiple correlated sites might demand the acquisition of higher-quality experimental data as well as more optimized fitting procedures compared with those assayed hereby for a reliable extraction of the parameters involved. Alternatively, however, QUICSY might provide a relatively straightforward experimental confirmation of parameters as estimated by other means (e.g., DFT calculations). In this regard, it shows some parallels with static 2D nutation line shape experiments that have been proposed in the literature.^68,69^

## 5 Conclusions and Outlook

This study discussed 2D QUICSY, an experiment with the potential to improve the resolution and information content of static NMR spectroscopy on half-integer quadrupoles. This type of correlations should thus find usefulness in cases characterized by a large second-order broadening, which render MAS less effective and are best measured under static conditions. The approach is aimed at exploiting the proportionality between the anisotropic broadenings of two isotopes belonging to the same element. The defining difference among these isotopes arises from different nuclear quadrupole and magnetic moments, which will shift the overall center of the patterns and scale their anisotropies. Calculations showed that 2D QUICSY spectra quickly gained complexity when considering multiple magnetically inequivalent sites endowed with sizable chemical shift anisotropies. The ensuing correlations led to off-diagonal patterns even for single sites. Sequences based on CP transfers were utilized to test these experiments on compounds, focusing on the ^85^Rb/^87^Rb isotope pair as the paradigm. Experimental results validated QUICSY’s ability to convey information on the size and relative orientations of the quadrupolar and chemical shift interaction tensors. The experiments also demonstrated that straightforward analytical 2D calculations that assumed ideal polarization transfers presented a good framework to describe and match the experimental data. From all of this, we conclude that the use of such correlation experiments could also yield an understanding of the structure of new compounds with unknown parameters. Numerous potential developments could be imparted based on the basic experiments performed here. The achievable resolution could be improved over the one shown, which was limited by a low SNR and the rapid ensuing decay of the signal in the indirect domain into noise; combining this experiment with hyperpolarization methods could hence be beneficial. Variations of the sequences that combine broadband excitations as well as broadband polarization transfers, including swept pulses (Figure S4), are also under study. Moreover, for species for which both isotopes possess low gyromagnetic ratios, such as ^35,37^Cl, where direct polarization transfer is expected to present a larger challenge, sequences mediated by protons as a source of spin diffusion and polarization transfer are also being considered.

## Supporting Information Available

Tensor orientational transformations used in this work; additional experimental details and data related to the QUICSY experiments, processing insight, and a variety of simulations and the scripts used to derive these data (PDF)
