# Supplementary material for: Quadrupolar Isotope-Correlation Spectroscopy in Solid-State NMR
Source: J Phys Chem C Nanomater Interfaces. 2022 May 17;126(22):9386–95. doi: 10.1021/acs.jpcc.2c00578 (PMC9189920; doi:10.1021/acs.jpcc.2c00578)
Supplement: Supplementary file 1 — jp2c00578_si_001.pdf [file jp2c00578_si_001.pdf]

Supporting information for

Quadrupolar Isotope-Correlation Spectroscopy in Solid State NMR

Tamar Wolf , Michael J. Jaroszewicz and Lucio Frydman*

Department of Chemical and Biological Physics, Weizmann Institute of Science, Rehovot 7610001, Israel

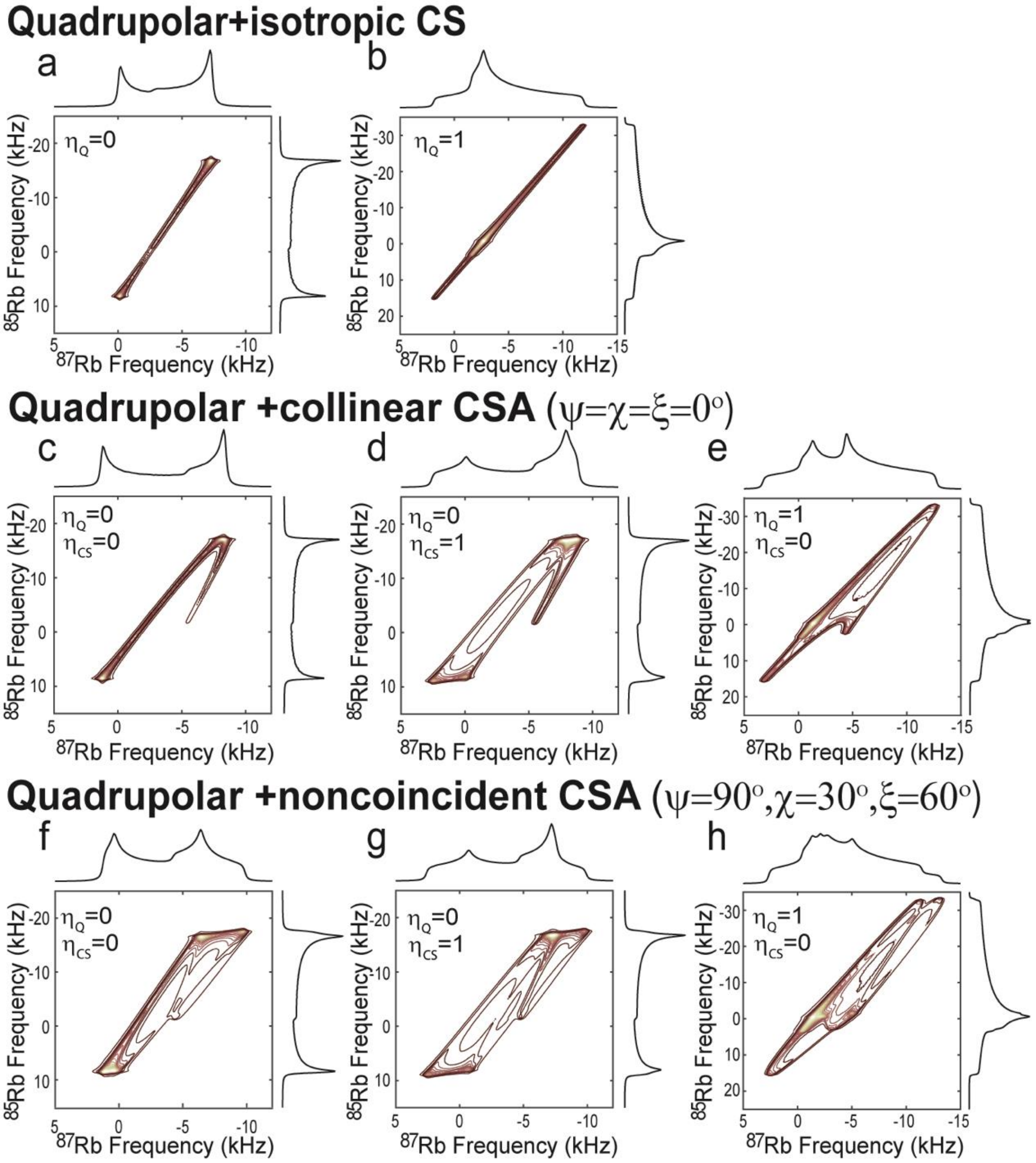

Figure S1. Analytically simulated 2D QUICSY correlation spectra between ^{85}Rb and ^{87}Rb at 14T according to Equations (1)-(3), showing how parameters affect the experiment's site resolution. In all panels C_Q=3.3 MHz and δ_{iso} =-13.7ppm for ^{87}Rb ($\omega_0 = 196.26$ MHz). For ^{85}Rb, the equivalent

scaled C_Q was used. (a-b) Quadrupolar and isotropic chemical shift only, with (a) $\eta_Q = 0$ and (b) $\eta_Q = 1$. In both cases a narrow ridge is preserved. (c-e) Addition of a collinear CSA tensor with a shielding anisotropy of δ_{aniso} =-13.8ppm. (c-e) Effects of adding η_Q and η_{CS}. When these are zero, a narrow correlation is still maintained, albeit with a hook-like shape. Lack of axial symmetry for both tensors, even if still collinear, results in some loss in resolution. This is only exacerbated when the tensors are noncoincident, as shown in (f-h) with a relative orientation of the CSA and quadrupolar tensors of $\psi = 90^\circ$, χ=30° and ξ=60°.

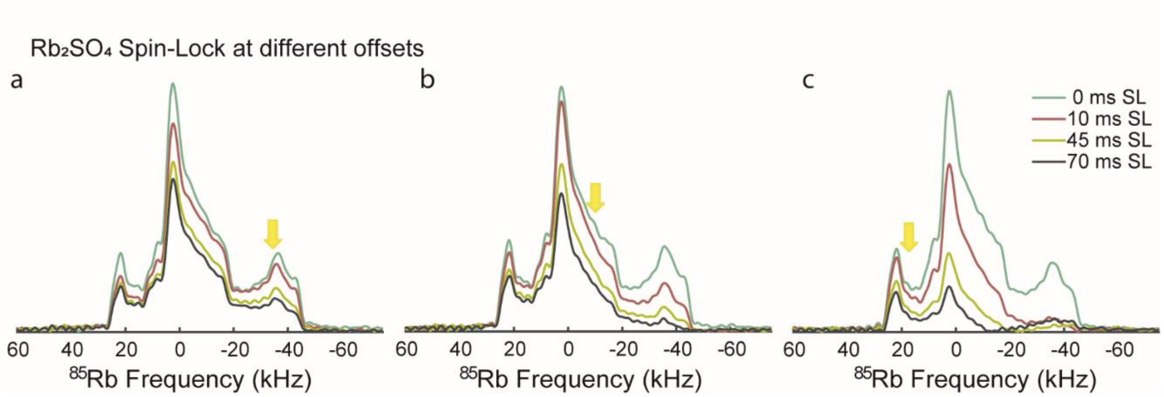

Figure S2. ^{85}Rb CT line shape and intensity for Rb_2SO_4 as a function of spin-lock length (0-70 ms), collected using an RF amplitude of ca. 26 kHz at three different transmitter frequency offsets: (a) -35 kHz (b) -10 kHz and (c) 18 kHz, marked by the yellow arrows. The sequence is depicted in figure 4b-c, and the echo acquisition time used was 0.64 ms, with a dead time of 37 μs before and after each π pulse.

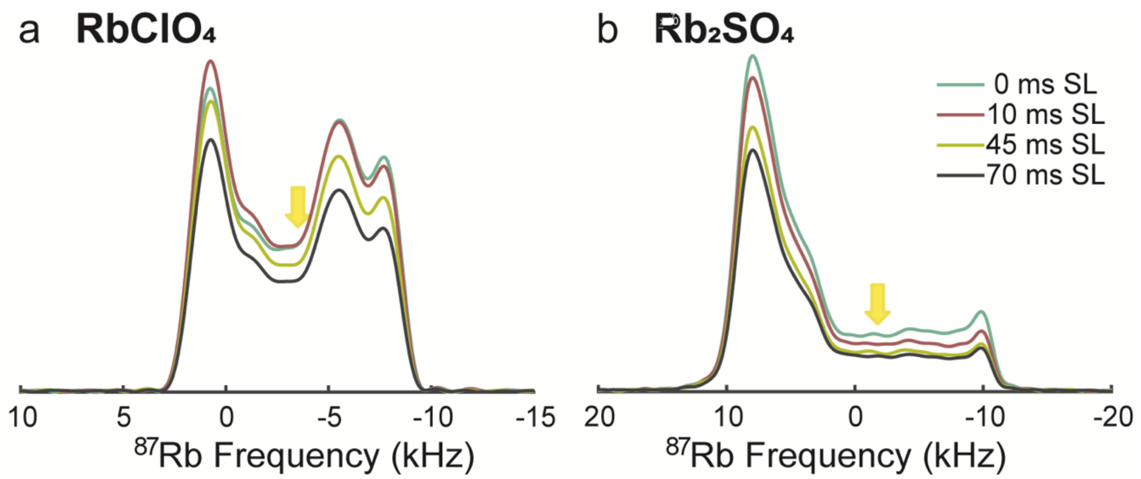

Figure S3. ^{87}Rb CT line shape and intensity as a function of spin-lock length (0-70 ms) using an RF amplitude of ca. 57 kHz for $RbClO_4$ (a) and Rb2SO$_4$ (b). The transmitter offset frequency is marked by the yellow arrows. The sequence is depicted in figure 4b-c, and the echo acquisition time used was 1.28 ms for both $RbClO_4$ and Rb_2SO_4, with a dead time of 7-8 μs before and after each π pulse.

In addition to a conventional CP, this study also considered a variation of CP where the spin lock pulse on the ^{85}Rb channel was replaced by WURST pulse (WCP), followed by a WURST-CPMG (WCPMG) acquisition block[4] (Figure S4a-b). The purpose of this variation was to ensure broadband polarization transfer from the ^{85}Rb channel. This sequence is similar in nature to BRAIN-CP-WCPMG[5], but involves the reversal of the WURST pulse and spin lock. Admittedly, this method still requires a spin-lock on ^{87}Rb. In order to incorporate a t_1-encoding for the 2D experiment, it is necessary to add an excitation block (Figure S4b), which is expected to lower the SNR of this experiment by a factor of ~2. The results of a 1D CP-CPMG experiment including both (±1) SQCs on Rb_2SO_4, were almost identical in SNR and line shape to those of a 1D WCP-WCPMG experiment (Figure S4c-d). For the constant-time experiment at t_1=0 (maximal signal) the SNR dropped by a factor of 2-3 for the WCP-WCPMG experiment, whereas it remained similar for the CP experiment (due to the lack of flip-back pulse). Due to this difference in signal, we preferred to acquire 2D CP-CPMG experiments at 3 different offsets and sum them. However, the WCP-WCPMG sequence could be of use for wider patterns, where the excitation block in Fig. S4b, could easily be converted to swept-pulses.

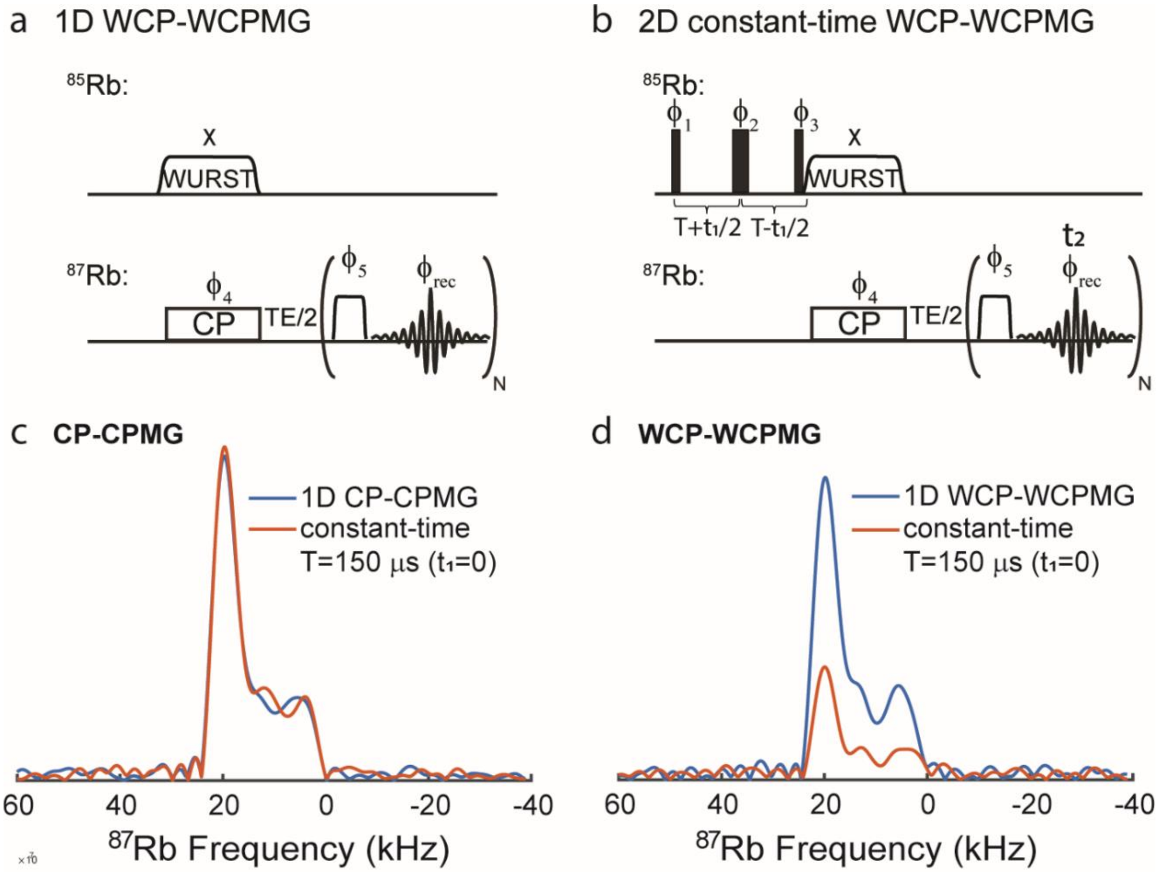

Figure S4. Swept WURST CP (WCP) sequences assayed in this study. (a) a WCP block, followed by WURST-CPMG acquisition (1D WCP-WCPMG). The phase-cycling was similar to 1D-CP-CPMG in Fgure 4a. To filter out thermal ^{87}Rb polarization, the frequency-sweep direction of the WURST pulse was alternated each scan, which resulted in the opposite sign of the coherence transferred to ^{87}Rb. (b) 2D constant-time WCP-WCPMG with an evolution period T, and a flip-back $\pi/2$ pulse before the swept contact pulse. This sequence employs the same phase-cycling as in the 2D constant time CP-CPMG, except ϕ_3 is the phase of the flip-back pulse. (c-d) Comparison between CP-CPMG (c) and WCP-WCPMG (d) experiments on Rb_2SO_4. Both are plotted in the same scale in the y-axis. In all experiments a DFS pulse was added to enhance the ^{85}Rb signal. For both cases the constant-time experiment (red) was measured with 4 times more scans than the regular 1D experiment (blue), and the resulting spectrum was normalized by $\sqrt{4} = 2$ in order to be comparable in SNR. (c) 1D CP-CPMG (Figure 4a), compared to a constant-time experiment at t_1=0 (figure 4b). The contact time was 65 ms. (d) 1D WCP-WCPMG and a constant time experiment at t_1=0. The contact time was 70 ms.

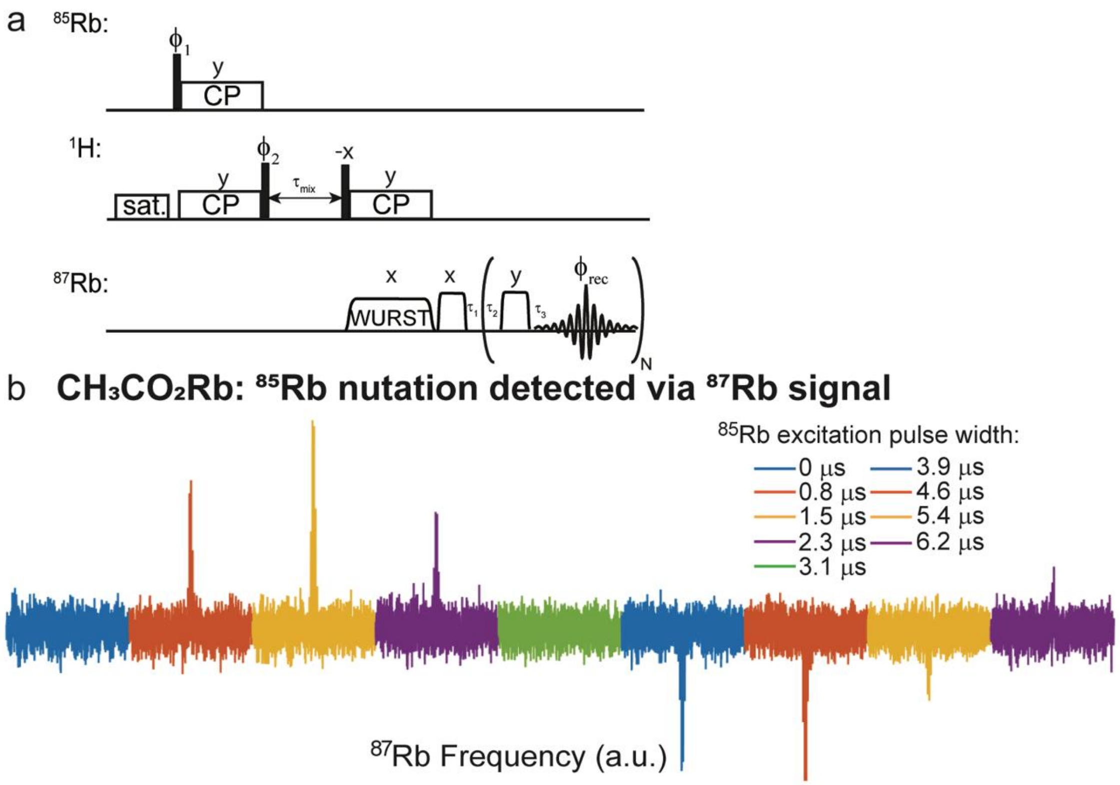

Figure S5. (a) Version of a ^{1}H-mediated QUICSY correlation sequence used in this study, incorporating a CP step from ^{85}Rb to ^{1}H, following a mixing time to allow for spin-diffusion, a second BRAIN-CP from ^{1}H to ^{87}Rb with WCPMG acquisition[5]. A 4-step phase cycling was employed: $\phi_1 = x, x, -x, -x$, $\phi_2 = x. -x, x, -x$, $\phi_{rec} = x, -x, -x, x$. This phase cycling allowed the evolution of both (±1) SQC of ^{85}Rb. (b) 1D nutation experiment on rubidium acetate (CH_3CO_2Rb), showing ^{87}Rb spectra as function of a variable flip-angle on the ^{85}Rb channel. The phased WCPMG signal (presented as spikelets) of ^{87}Rb followed the ^{85}Rb nutation.

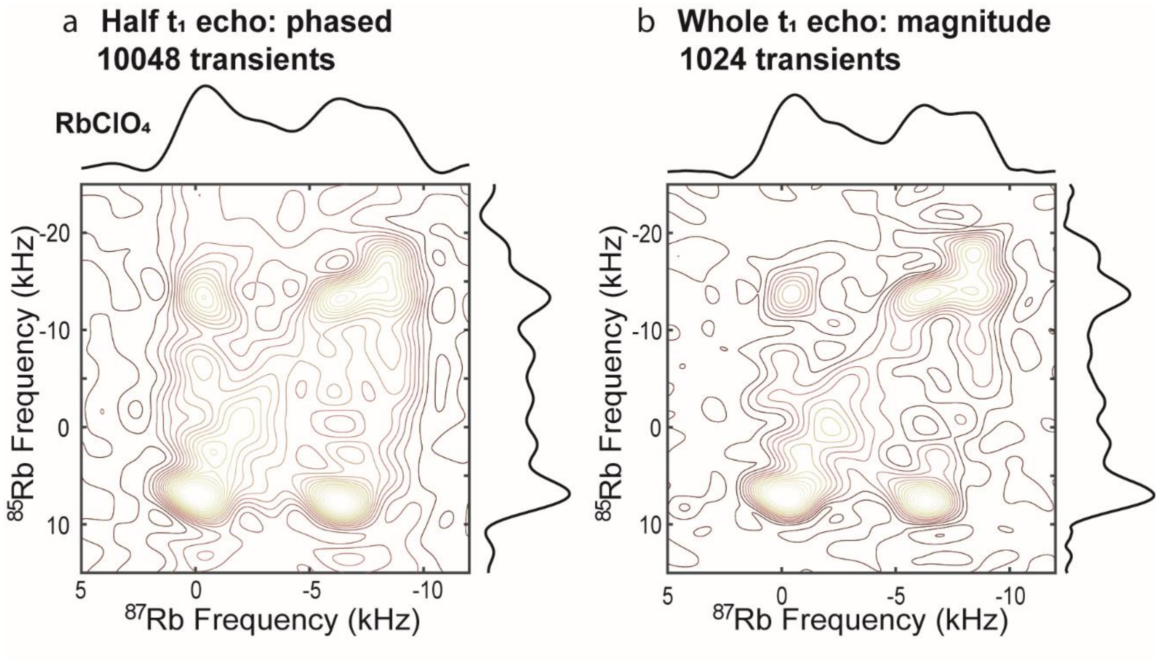

Figure S6 Experimental QUICSY spectra of $RbClO_4$ with whole-echo acquisition. (a) Same raw data as in Figure 6a, but processing only the echo pathway (positive t1s) using 12 t_1 increments and phasing the data (sw1=50 kHz). (b) Same parameters and processing as Figure 6a, but with ca. $1/10^{th}$ less transients. The data is presented in magnitude. The SNR is lower, but most of the information is preserved (13.5 hr acquisition time).

The frequency-stepped 2D QUICSY acquisitions on Rb_2SO_4 resulted in small intensity jumps following the summation of the spectra. These jumps occurred in the points of overlap between the ranges covered by the spectral width in the indirect dimension (sw1). For Figure S7 and figure 6, the overlap occurs outside the range [-50 30] kHz due to setting sw1=150 kHz. In order to reduce experimental time, it is possible to set sw1=100 kHz or even lower. Then, some of the overlap will occur in this range, resulting in small intensity jumps marked by arrows (Figure S8). However, these can be easily corrected in the projection along F1, and are barely noticeable in the 2D contour. Another way to reduce experimental time, is to acquire a slightly asymmetric echo in the t1 domain. In this case, the dispersive component might not be zero, but the effect on the 2D contour is barely visible (Figure S8).

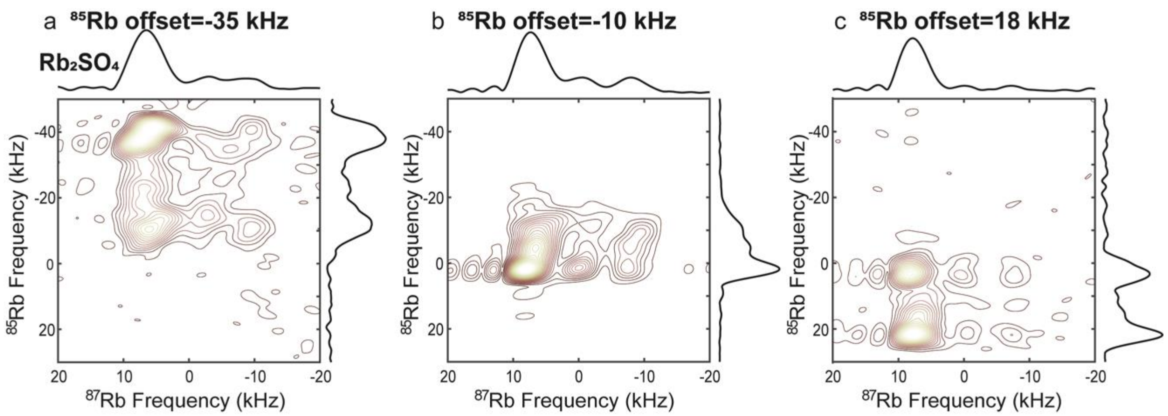

Figure S7 2D QUICSY spectra of Rb_2SO_4 acquired at three different ^{85}Rb offsets: -35 kHz (a), -10 kHz (b) and 18 kHz (c). A CP contact-time of 70 ms was used, and an RF amplitude of ca. 30 kHz on the ^{85}Rb channel and 45 kHz on the ^{87}Rb channel. Whole echo acquisition was carried out, with a CPMG echo acquisition time TE=320 μs (7 μs dead time before and after each π pulse, sw=100 kHz) and a total of 49 t_1 increments constituting a symmetric t_1-echo (sw1=150 kHz). Notice (by the noise) that the 2D contours and projections of (a)-(c) are not presented in the same intensity scale. The 2Ds in (a) and (c) are presented with 20 contour levels, whereas the 2D in (b) is presented with 30 contour levels.

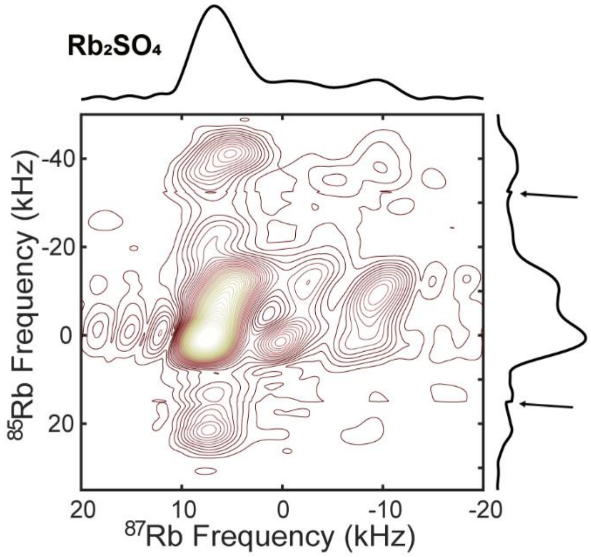

Figure S8 2D QUICSY spectra of Rb_2SO_4 acquired at almost the same conditions as Figure S7. The main difference was setting sw1=100 kHz and a total of 22 t_1 increments (not a fully symmetric t_1-echo). The data 2D is presented in magnitude.

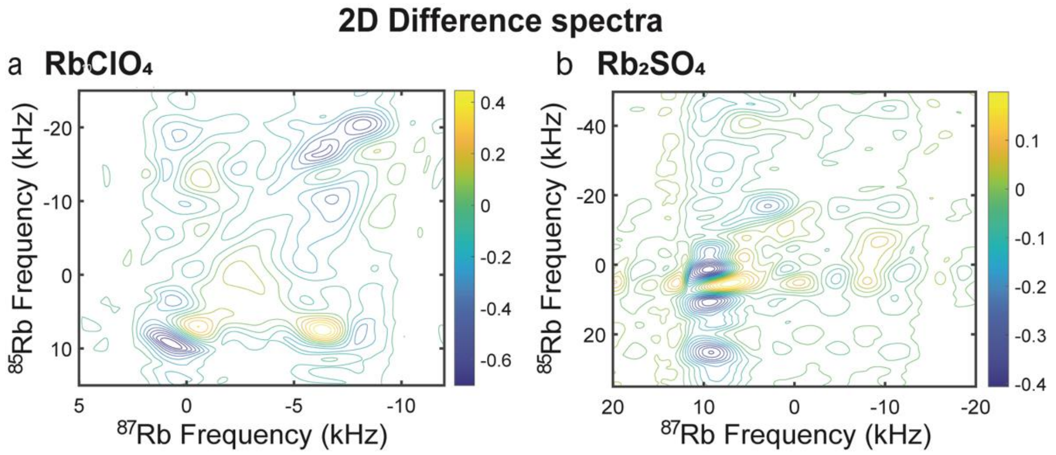

Figure S9 Difference spectra arising between the experimental data and analytical calculations based on the parameters depicted in figure 6. Both subtracted 2D datasets were normalized to a maximum of 1 before taking their difference, and then subtracted over the same range. Since the experimental data is in magnitude, the analytical calculated was also subtracted in magnitude mode. (a) Difference spectrum for the $RbClO_4$ data in figure 6a-b (c) Difference spectrum for the Rb_2SO_4 data in figure 6c-d.

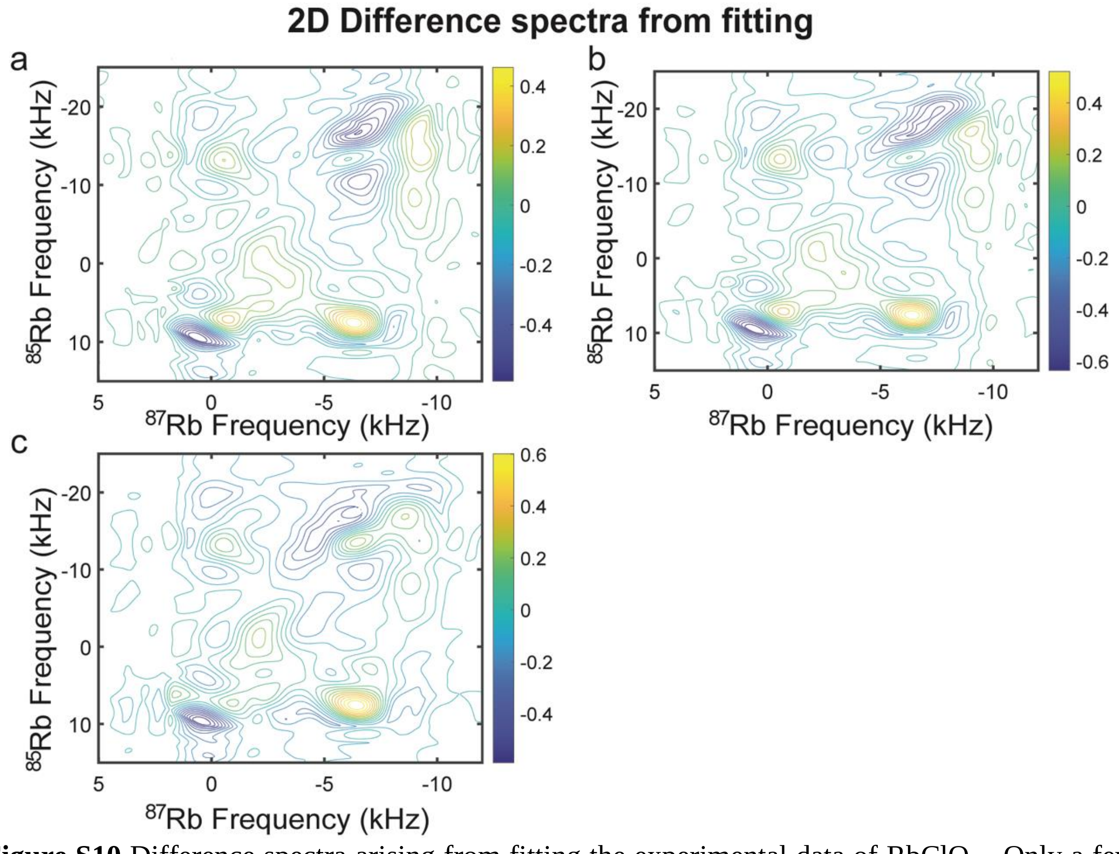

Figure S10 Difference spectra arising from fitting the experimental data of $RbClO_4$. Only a few parameters were fitted at a time, all based on the analytical expressions for the NMR frequencies. A numerical optimization function was used to minimize the residual norm based on an initial guess (literature results) and a range (with upper and lower bound) for each parameter. (a) Difference spectrum based on fitting C_Q, η_Q, δ_{aniso} and η_{CS}. The result of the best fit was C_Q=3.25 MHz, $\eta_Q =$

0.17, $\delta_{aniso} = -11.5$ ppm and $\eta_{CS} = 0.56$. The upper and lower bounds for each respective parameter were ([3.25, 3.35], [0.17, 0.25], [-14.8 , -11.5], [0.56, 0.66]). (b) Difference spectrum based on fitting C_Q, η_Q, δ_{aniso}, η_{CS}, ϕ, κ and ζ. The result of the best fit was C_Q=3.29 MHz, $\eta_Q = 0.2$, $\delta_{aniso} = -11.5$ ppm, ϕ=–120˚, κ=83.8˚ and ζ=33.4˚. The upper and lower bounds for each respectively were ([3.29, 3.35], [0.17, 0.23], [-14.8 , -11.5], [-120˚, -80˚], [60˚, 120˚], [20˚, 90˚]). (c) Difference spectrum based on fitting ψ, χ, and ξ. The result of the best fit was , ψ=164˚, χ=35˚, ξ=56˚. The upper and lower bounds for each respectively were ([0˚, 180˚], [0˚, 90˚], [0˚, 90˚]).

Table S1. Summary of literature parameters of $RbClO_4$

C_Q	η_Q	δ_{aniso}	η_{CS}	δ_{iso}	ψ	χ	ξ	Approach	Ref
3.30±0.04	0.21±0.03	13.8±1.5	0.61±0.24	-13.7±0.6	94±14	28±4	87±4	single- crystal	(6)
3.29±0.05	0.20±0.03	13.5±1.0	0.32±0.20	-13.1±0.3	98±15	34±5	69±20	MAS	(2)
3.19±0.10	0.16±0.04	11.7[a]	0.25±0.04	-3.8	62±4	81±1	58±4	static	(7)
3.49	0.17	-9.34[b]	0.63	-	29	-27.5	86.5	Static and MAS	(8)

[a]For the values given by reference (5), δ_{aniso} was calculated from the $\Delta\sigma$ there given based on the relation $\delta_{aniso} = \frac{2}{3}\Delta\sigma$.[4]

[b]The notation $\Delta\sigma$ used in this publication refers to the δ_{aniso} used in our study. We converted δ_{aniso} from Hz to ppm.

Table S2. Summary of literature parameters of Rb_2SO_4.

Site	C_Q	η_Q	δ_{aniso}	η_{CS}	δ_{iso}	ψ	χ	ξ	Approach	Ref
1	2.72±0.03	0.93±0.03	2.7±0.9	0.26±0.62	42.6±0.3	76±89	17±13	110±37	single - crystal	**(6)**
2	5.29±0.05	0.12±0.03	-25±3	0.54±0.3	15.5±1.5	9±26	37±5	270±22		
1	2.65±0.01	0.99±0.01	-9±4[a]	0.36±0.33	42.3±0.1	-6±25	62±22	0±88	MAS	(9)
2	5.31±0.03	0.11±0.01	-23±3[a]	0.55±0.68	15.8±0.2	21±29	36±8	77±44		
1	2.6	1	-	-	40	-	-	-	SAS	(10)
2	5.3	0.1	-	-	16	-	-	-		

[a]For the values given by reference (7), δ_{aniso} was calculated from the $\Delta\sigma$ there given based on the relation $\delta_{aniso} = \frac{2}{3}\Delta\sigma$.[4]

Mathematica script used in this study to calculate the various frame transformations:

```
Ra = {{cos[α], sin[α], 0}, {-sin[α], cos[α], 0}, {0, 0, 1}}
Rb = {{cos[β], 0, -sin[β]}, {0, 1, 0}, {sin[β], 0, cos[β]}}
R = Rb . Ra
RI = Transpose[R]
Vp = DiagonalMatrix[{V11, V22, V33}]
VL = R . Vp . RI
VLAB1 = -VL[[1,3]]^2- VL[[2,3]]^2 + (1/2)*((1/4)*(VL[[1,1]]-
VL[[2,2]])^2 + VL[[1,2]]^2)
Simplify[VLAB1]
```

```
RE = {{cos[e], sin[e], 0}, {-sin[e], cos[e], 0}, {0, 0, 1}}
Rf = {{cos[f], 0, -sin[f]}, {0, 1, 0}, {sin[f], 0, cos[f]}}
Rc = {{cos[c], sin[c], 0}, {-sin[c], cos[c], 0}, {0, 0, 1}}
RQtQ = Rc . Rf . RE
RQtQT = Transpose[RQtQ]
VL2 = R . RQtQ . Vp . RQtQT . RI

VLAB2 = - VL2 [[1,3]]^2 - VL2 [[2,3]]^2 + (1/2)*((1/4)*( VL2 [[1,1]] -
VL2 [[2,2]])^2 + VL2 [[1,2]]^2)

Simplify[VLAB2]

Dp = DiagonalMatrix[{D11, D22, D33}]

R1 = {{cos[a], sin[a], 0}, {-sin[a], cos[a], 0}, {0, 0, 1}}
R2 = {{cos[b], 0, -sin[b]}, {0, 1, 0}, {sin[b], 0, cos[b]}}
R3 = {{cos[g], sin[g], 0}, {-sin[g], cos[g], 0}, {0, 0, 1}}
RCStQ = R3 . R2 . R1
RCStQT = Transpose[RCStQ]
DL = R . RCStQ . Dp . RCStQT . RI
DLAB1 = (1/3)*(2* DL [[3,3]] - (DL [[2,2]] + DL [[1,1]]))
Simplify[DLAB1]

DL2 = R . RQtQ . RCStQ . Dp . RCStQT . RQtQT . RI
DLAB2 = (1/3)*(2* DL2 [[3,3]] - (DL2 [[1,1]] + DL2 [[2,2]]))
Simplify[DLAB2]
```

In these expressions VLAB1 is the orientation-dependent term of a single quadrupolar tensor in the lab frame as written in equation A.11-A.12 of the appendix (VLAB1=$A^{Q}_{2,-1}A^{Q}_{2,1}+\frac{1}{2}A^{Q}_{2,-2}A^{Q}_{2,2}$). VLAB2 is the orientation dependence of a second magnetically inequivalent quadrupolar tensor with an additional transformation as specified in A.23. DLAB1 is the orientation-dependent term of a chemical shift tensor in the presence of a quadrupolar tensor as specified in A.20-A.21 (DLAB1=$\sqrt{\frac{2}{3}}A_{20}$). DLAB2 is the orientation dependence of a second magnetically inequivalent CSA tensor with an additional transformation as specified in A.24.

Appendix: Tensors, orientations, and transformations considered in this work

As a number of different parametric definitions regarding coupling parameters and rotations of Hamiltonians have been given in the solids NMR literature, we define in a short Appendix the conventions used in this work.

All interactions were represented by second-rank Cartesian tensors[11]. The quadrupolar interaction was described by:

$$H_Q = \frac{eQ}{2I(2I-1)\hbar}\vec{I}\cdot\overleftrightarrow{V}\cdot\vec{I} \qquad \text{A.1}$$

and the chemical shift interaction by:

$$H_{cs} = \gamma\cdot\vec{I}\cdot\overleftrightarrow{\delta}\cdot\vec{B} \qquad \text{A.2}$$

In the principal axis system (PAS) each tensor will be characterized by its three principal values: $[A_{xx}]^P, [A_{yy}]^P, [A_{zz}]^P$, where we use the convention $|[A_{zz}]^P| \geq |[A_{xx}]^P| \geq \left|[A_{yy}]^P\right|$. The quadrupolar tensor $\overleftrightarrow{V}$ is traceless and therefore does not have an isotropic value; it is then characterized by only two parameters[42]:

$$C_Q = \frac{e^2qQ}{\hbar} \text{ - the nuclear quadrupolar coupling constant.} \qquad \text{A.3}$$

$$\eta_Q = \frac{[V_{yy}]^P-[V_{xx}]^P}{[V_{zz}]^P} \text{- asymmetry parameter.} \qquad \text{A.4}$$

The chemical shift tensor is described (in ppm) by the following three parameters[13,15]:

$$\delta_{iso} = \frac{1}{3}\left([\delta_{xx}]^P + [\delta_{yy}]^P + [\delta_{zz}]^P\right) \text{ – isotropic shift}$$

A.5

$$\delta_{aniso} = [\delta_{zz}]^P - \delta_{iso} \text{ – shielding anisotropy}$$

A.6

$$\eta_{CS} = \frac{[\delta_{yy}]^P - [\delta_{xx}]^P}{\delta_{aniso}} \text{ – asymmetry parameter}$$

A.7

with $|[\delta_{zz}]^P - \delta_{iso}| \geq |[\delta_{xx}]^P - \delta_{iso}| \geq \left|[\delta_{yy}]^P - \delta_{iso}\right|$ and $0 \leq \eta \leq 1$.

Typically, one needs to transform the Hamiltonian from its PAS to various frames,[12,11] including the laboratory frame where the secular approximation is taken. If spin anisotropies are described in terms of irreducible tensors involving products of spatial and

spin spherical tensors A_{lm}, T_{lm}, then the quadrupolar interaction involve second rank components:

$$H_Q = \frac{\omega_Q}{3}\sum_{m=-2}^{2}(-1)^m A_{2,m}^Q T_{2,-m}^Q,$$

A.8

where $\omega_Q = \frac{3C_Q}{2I(2I-1)}$ and the $\{A_{2,m}\}$ are described in terms of the Cartesian tensor components as [12]:

$A_{2,0}^Q = 3\sqrt{1/6}V_{zz}$; $A_{2,\pm1}^Q = \mp V_{xz} - iV_{yz}$ and $A_{2,\pm2}^Q = \frac{1}{2}(V_{xx} - V_{yy}) \pm iV_{xy}$

A.9

Second-order perturbation theory is then necessary to calculate the central-transition frequency of half-integer spins, which is[12]:

$$\omega_Q^{(2)} = -\frac{1}{18\omega_0}\left[\frac{3C_Q}{2I(2I-1)}\right]^2 \cdot \left(A_{2,-1}^Q A_{2,1}^Q + \frac{1}{2}A_{2,-2}^Q A_{2,2}^Q\right) \cdot (3 - 4I(I+1))$$

A.10

The transformation of the quadrupolar tensor from its PAS to the laboratory frame can be expressed as

$$V_Q^{LAB} = R_Q^{PL} \cdot V_Q^P \cdot R_Q^{PL^T} \qquad \text{A.11}$$

where $R_Q^{PL}(\alpha, \beta, \gamma = 0^\circ)$ is an Euler rotation matrix. Once rotated, the elements of the Cartesian tensor V_Q^{LAB} can then be used to calculate central-transition frequency according to equation A.9, leading to:

$$A_{2,-1}^Q A_{2,1}^Q + \frac{1}{2}A_{2,-2}^Q A_{2,2}^Q = -{V_{Q1,xz}^{LAB}}^2 - {V_{Q1,yz}^{LAB}}^2 + \frac{1}{2}\left(\frac{1}{4}(V_{Q1,xx}^{LAB} - V_{Q1,yy}^{LAB})^2 + {V_{Q1,xy}^{LAB}}^2\right)$$

A.12

Substituting this in equation A.10:

$$\omega_Q^{(2)} = -\frac{1}{18\omega_0}\left[\frac{3C_Q}{2I(2I-1)}\right]^2 \left(-{V_{Q1,xz}^{LAB}}^2 - {V_{Q1,yz}^{LAB}}^2 + \frac{1}{2}\left(\frac{1}{4}(V_{Q1,xx}^{LAB} - V_{Q1,yy}^{LAB})^2 + {V_{Q1,xy}^{LAB}}^2\right)\right)(3 - 4I(I+1))$$

A.13

This leads to Equation 2 in the main text,

$$\omega_Q^{(2)} = -\frac{1}{6\omega_0}\left[\frac{3C_Q}{2I(2I-1)}\right]^2 \left[I(I+1) - \frac{3}{4}\right] \cdot [A(\alpha, \eta_Q)\cos^4\beta + B(\alpha, \eta_Q)\cos^2\beta + C(\alpha, \eta_Q)]$$

A.14

with[12]

$$A(\alpha, \eta) = -\frac{27}{8} + \frac{9}{4}\eta_Q \cos 2\alpha - \frac{3}{8}(\eta \cos 2\alpha)^2. \qquad \text{A.15}$$

$$B(\alpha,\eta) = \frac{30}{8} - \frac{1}{2}\eta_Q^2 - 2\eta_Q \cos 2\alpha + \frac{3}{4}(\eta \cos 2\alpha)^2. \qquad \text{A.16}$$

$$C(\alpha,\eta) = -\frac{3}{8} + \frac{1}{3}\eta_Q^2 - \frac{1}{4}\eta_Q \cos 2\alpha - \frac{3}{8}(\eta \cos 2\alpha)^2. \qquad \text{A.17}$$

A similar calculation for the chemical shift Hamiltonian leads to[12,13]:

$$H_{CS} = \gamma\delta_{iso}B_z I_z + \sqrt{\frac{2}{3}}\gamma\delta_{aniso}\sum_{m=-2}^{2}(-1)^m A_{2,m}^{CS} T_{2,-m}^{CS}.$$

A.18

For the chemical shift first-order perturbation theory suffices, leading to[41,12]:

$$\omega_{CS} = \omega_0(\delta_{iso} + \sqrt{\frac{2}{3}}\delta_{aniso}A_{2,0}^{CS}) \qquad \text{A.19}$$

which is Equation (3) in the main text. In the present study, the orientation-dependence of the chemical shift was calculated by first transforming this tensor from its PAS into the PAS of the quadrupolar interaction, and then onto the lab frame. This transformation is given in Cartesian components by:

$$\delta^{LAB} = R_Q^{PL} \cdot R^{CE} \cdot \delta^{PAS} \cdot R^{CE} \cdot R_Q^{PL^T} \qquad \text{A.20}$$

Here R^{CE}, the rotation matrix from the PAS of the CSA tensor to the PAS of the EFG tensor, is described by the Euler angles ψ, χ, ξ: $R^{CE} = R(\psi, \chi, \xi)$, and R_Q^{PL} is as described above. The elements of the Cartesian tensor δ^{LAB} can then be used to calculate the chemical shift frequency according to:

$$\sqrt{\frac{2}{3}}A_{2,0}^{CS} = \frac{1}{3}(2\delta_{zz}^{LAB} - \delta_{xx}^{LAB} - \delta_{yy}^{LAB}) = (\frac{3\cos^2\chi - 1}{2} - \frac{1}{2}\eta_\sigma \cdot \sin^2\chi \cos 2\psi) \cdot \left(\frac{3\cos^2\beta - 1}{2}\right) - (3 + \eta_\sigma \cdot \cos 2\psi) \cdot 0.25 \cdot \sin 2\chi \cdot \sin 2\beta \cdot \cos(\xi + \alpha) + 0.5\eta_\sigma \cdot \sin\chi \cdot \sin 2\psi \cdot \sin 2\beta \cdot \sin(\xi + \alpha) + (0.75\sin^2\chi - 0.25\eta_\sigma \cdot (\cos^2\chi + 1) \cdot \cos 2\psi) \cdot \sin^2\beta \cdot \cos(2\alpha + 2\xi) + 0.5\eta_\sigma \cdot \cos\chi \cdot \sin 2\psi \cdot \sin^2\beta \cdot \sin(2\alpha + 2\xi)$$

A.21

Note that when ($\psi = 0, \chi = 0, \xi = 0$), equation A.21 reduces to

$$\sqrt{\frac{2}{3}}A_{2,0}^{CS} = \frac{1}{2}\left((3\cos^2\beta - 1) - \eta_\sigma \cos(2\alpha)\sin^2\beta\right)$$

A.22

as expected.

The simulations in the main text called for accounting for a second, chemically equivalent but magnetically inequivalent site, related to one another by a crystal-imposed transformation. The anisotropies of this second site can be accounted for by an additional set of transformations taking the second nucleus' interactions into the quadrupolar PAS of the first nucleus –and proceeding thereafter as above. Denoting for simplicity the second site by a subindex 2, the transformation of its Cartesian EFG tensor from its PAS to the lab frame can be described as:

$$V_{Q2}^{LAB} = R_{Q1}^{PL} \cdot \mathrm{R}_{\mathrm{Q2}}^{\mathrm{P2P1}} \cdot V_{Q2}^{P} \cdot \mathrm{R}_{\mathrm{Q2}}^{\mathrm{P2P1}^{\mathrm{T}}} \cdot R_{Q1}^{PL^{T}} \quad \text{A.23}$$

Here $V_{Q2}^{P} = V_{Q1}^{P}$, the tensor of site 1, yet the PAS orientations themselves are not aligned. $\mathrm{R}_{\mathrm{Q2}}^{\mathrm{P2P1}}$ describes the transformation from the PAS of V_{Q2}^{P} to the PAS of V_{Q1}^{P} with the Euler angles ϕ, κ, ζ: $\mathrm{R}_{\mathrm{Q2}}^{\mathrm{P2P1}} = R(\phi, \kappa, \zeta)$, and R_{Q1}^{PL} is as described above in Equation A.11. With this transformation, the elements of the Cartesian tensor V_{Q2}^{LAB} can then be substituted in the central-transition frequency as in Equation A.13. Likewise, the CSA tensor orientation of the second magnetically inequivalent site can be calculated by adding an additional transformation R^{CE}:

$$\delta_{2}^{LAB} = R_{Q1}^{PL} \cdot \mathrm{R}_{\mathrm{Q2}}^{\mathrm{P2P1}} \cdot R^{CE} \cdot \delta^{PAS} \cdot R^{CE} \cdot \mathrm{R}_{\mathrm{Q2}}^{\mathrm{P2P1}^{\mathrm{T}}} \cdot R_{Q1}^{PL^{T}} \quad \text{A.24}$$

The elements of the Cartesian tensor δ_{2}^{LAB} were then substituted to the chemical shift frequency in the same manner as Equation A.21.

For sites related by crystallographic symmetry, the $\mathrm{R}_{\mathrm{Q2}}^{\mathrm{P2P1}}(\phi, \kappa, \zeta)$ matrices were calculated according to reference 14, as:

$$\mathrm{R}_{\mathrm{Q2}}^{\mathrm{P2P1}}(\phi, \kappa, \zeta) = DCM \cdot DCM \cdot M$$

A.25

Here DCM is the direction cosine matrix describing the quadrupolar tensor in the crystal-frame (e.g., Table 2 in reference 14); i.e., it is a transformation matrix relating the crystal-frame to the PAS of the quadrupolar tensor. M, on the other hand, describes a reflection matrix that relates the two magnetically inequivalent sites in the crystal frame. The reflection matrix may also be part of a glide plane that relates the two magnetically nonequivalent sites (the translation component of the glide plane does not affect the observed NMR frequency). In the case of $RbClO_4$, $M = \begin{bmatrix} -1 & 0 & 0 \\ 0 & 1 & 0 \\ 0 & 0 & -1 \end{bmatrix}$, whereas for

Rb_2SO_4 $M = \begin{bmatrix} -1 & 0 & 0 \\ 0 & -1 & 0 \\ 0 & 0 & 1 \end{bmatrix}$.[14] The first multiplication of the tensor by $DCM \cdot M$ transforms V_{Q2}^{P} from its PAS to the crystal frame, and the following multiplication by DCM, transforms it back from the crystal-frame to the PAS of V_{Q1}^{P}. The Euler angles between the two different PASs found based on the literature DCM[14] and equation A.25 were $\phi = -112°, \kappa = 103°, \zeta = 24°$ for $RbClO_4$. For Rb_2SO_4, two sets of angles were found (one for each site): $\phi_1 = 84°, \kappa_1 = 174°, \zeta_1 = -100°$ and $\phi_2 = 39°, \kappa_2 = 43°, \zeta_2 = 97°$.

References

(1) Skibsted, J.; Nielsen, N. C.; Bildsoe, H.; Jakobsen, H. J. Magnitudes and Relative Orientation of Vanadium-51 Quadrupole Coupling and Anisotropic Shielding Tensors in Metavanadates and Potassium Vanadium Oxide (KV3O8) from Vanadium-51 MAS NMR Spectra. Sodium-23 Quadrupole Coupling Parameters for .Alpha.- and .Bet. *J. Am. Chem. Soc.* **1993**, *115* (16), 7351–7362. https://doi.org/10.1021/ja00069a038.

(2) Vosegaard, T.; Skibsted, J.; Bildsoe, H.; Jakobsen, H. J. Combined Effect of Second-Order Quadrupole Coupling and Chemical Shielding Anisotropy on the Central Transition in MAS NMR of Quadrupolar Nuclei. 87Rb MAS NMR of RbClO4. *J. Phys. Chem.* **1995**, *99* (27), 10731–10735. https://doi.org/10.1021/j100027a009.

(3) Fernandez, C.; Bodart, P.; Amoureux, J. P. Determination of 51V Quadrupole and Chemical Shift Tensor Orientations in V2O5 by Analysis of Magic-Angle Spinning Nuclear Magnetic Resonance Spectra. *Solid State Nucl. Magn. Reson.* **1994**, *3* (2), 79–91. https://doi.org/https://doi.org/10.1016/0926-2040(94)90026-4.

(4) O'Dell, L. A.; Schurko, R. W. QCPMG Using Adiabatic Pulses for Faster Acquisition of Ultra-Wideline NMR Spectra. *Chem. Phys. Lett.* **2008**, *464* (1), 97–102. https://doi.org/https://doi.org/10.1016/j.cplett.2008.08.095.

(5) Harris, K. J.; Lupulescu, A.; Lucier, B. E. G.; Frydman, L.; Schurko, R. W. Broadband Adiabatic Inversion Pulses for Cross Polarization in Wideline Solid-State NMR Spectroscopy. *J. Magn. Reson.* **2012**, *224*, 38–47. https://doi.org/https://doi.org/10.1016/j.jmr.2012.08.015.

(6) Vosegaard, T.; Skibsted, J.; Bildsøe, H.; Jakobsen, H. J. Quadrupole Coupling and Anisotropic Shielding from Single-Crystal NMR of the Central Transition for Quadrupolar Nuclei.87Rb NMR of RbClO4and Rb2SO4. *J. Magn. Reson. Ser. A* **1996**, *122* (2), 111–119. https://doi.org/https://doi.org/10.1006/jmra.1996.0186.

(7) Cheng, J. T.; Edwards, J. C.; Ellis, P. D. Measurement of Quadrupolar Coupling Constants, Shielding Tensor Elements and the Relative Orientation of Quadrupolar and Shielding Tensor Principal Axis Systems for Rubidium-87 and Rubidium-85 Nuclei in Rubidium Salts by Solid-State NMR. *J. Phys. Chem.* **1990**, *94* (2), 553–561. https://doi.org/10.1021/j100365a014.

(8) Wolf, T.; Jaroszewicz, M. J.; Frydman, L. Steady-State Free Precession and Solid-State NMR: How, When, and Why. *J. Phys. Chem. C* **2021**, *125* (2), 1544–1556. https://doi.org/10.1021/acs.jpcc.0c09696.

(9) Fernandez, C.; Amoureux, J. P.; Bodart, P. Electronic Shieldings and Electric Field Gradients in Rubidium Sulfate Studied by 87Rb MAS NMR. *J. Magn. Reson. Ser. A* **1995**, *113* (2), 205–209. https://doi.org/https://doi.org/10.1006/jmra.1995.1081.

(10) Shore, J. S.; Wang, S. H.; Taylor, R. E.; Bell, A. T.; Pines, A. Determination of Quadrupolar and Chemical Shielding Tensors Using Solid-state Two-dimensional NMR Spectroscopy. *J. Chem. Phys.* **1996**, *105* (21), 9412–9420. https://doi.org/10.1063/1.472776.
(11) Michael Mehring. *Principles of High-Resolution NMR in Solids*, 2nd ed.; Springer-Verlag, 1983.
(12) Man, P. P. Quadrupolar Interactions. *eMagRes* **2011**. https://doi.org/doi:10.1002/9780470034590.emrstm0429.pub2.
(13) Edén, M. Computer Simulations in Solid-State NMR. I. Spin Dynamics Theory. *Concepts Magn. Reson. Part A* **2003**, *17A* (1), 117–154. https://doi.org/10.1002/cmr.a.10061.
(14) Vosegaard, T.; Skibsted, J.; Bildsøe, H.; Jakobsen, H. J. Quadrupole Coupling and Anisotropic Shielding from Single-Crystal NMR of the Central Transition for Quadrupolar Nuclei.87Rb NMR of RbClO4and Rb2SO4. *J. Magn. Reson. Ser. A* **1996**, *122* (2), 111–119. https://doi.org/https://doi.org/10.1006/jmra.1996.0186.
(15) Levitt, M. H. The Signs of Frequencies and Phases in NMR. *J. Magn. Reson.* **1997**, *126* (2), 164–182. https://doi.org/https://doi.org/10.1006/jmre.1997.1161.
